# Family, school and individual characteristics associated with adolescents’ physical activity at school in Hong Kong: the iHealt(H) study

**DOI:** 10.1186/s12966-021-01085-z

**Published:** 2021-01-19

**Authors:** Alison Carver, Muhammad Akram, Anthony Barnett, Wendy Yajun Huang, Gemma Yang Gao, Robin R. Mellecker, Ester Cerin

**Affiliations:** 1 Mary MacKillop Institute for Health Research, Australian Catholic University, Level 5, 215 Spring Street, Melbourne, Victoria 3000 Australia; 2 Hong Kong Baptist University, Hong Kong, China; 3 Faculty of Education, The University of Hong Kong, Hong Kong, China; 4 School of Public Health, The University of Hong Kong, Hong Kong, China

**Keywords:** Household characteristics, Family, School, Adolescent, Mediation analysis, Physical activity, Asia

## Abstract

**Background:**

Adolescents (11–18-year-olds) are at risk of physical inactivity. There is limited knowledge of physical activity (PA) levels among adolescents in the school setting in Hong Kong. We developed and tested a novel theoretical model of how household/family characteristics, school-level social and physical environmental factors and individual adolescent’s characteristics impact on their school-based PA during and after school hours.

**Methods:**

Cross-sectional study participants were Hong Kong adolescents attending secondary school, paired with their parent/caregiver (*n* = 1299 dyads). Parents survey-reported on household/family characteristics, parental PA and rules related to PA. Adolescents survey-reported on school PA-friendly policy, PA equipment at school (combined to create PA-friendly index), social support for PA from peers, athletic ability, attitude to and enjoyment of PA. Adolescents self-reported their school-based PA during school hours (physical education; recess) and after school (sports teams/classes). Objectively-measured moderate-to-vigorous PA (MVPA) was measured using accelerometers for a sub-sample of adolescents (*n* = 588). Generalized additive mixed models were used to estimate how household/family-level and school-level factors, and adolescents’ individual characteristics were related to adolescents’ school-based PA in Hong Kong, and to identify potential mediators of these associations.

**Results:**

A complex network of potential pathways of influence on adolescents’ school-based PA was identified. Overall, most of the significant effects were indirect ones. However, there were far fewer significant pathways between household/family characteristics and objectively-measured MVPA at school than there were for self-reported PA at school. In fact, there were no indirect pathways between these variables and MVPA at school. Gender disparities among pathways were identified. For example, school PA-friendly index was significantly associated with MVPA after school only among girls (e^b^ = 1.06, 95%CI (1.02,1.12)).

**Conclusions:**

Key points of intervention identified by our study may be in the re-design of PE classes so that adolescents spend more time being physically active during these classes, and promotion of active play during recess. Further research measuring amount, intensity and location of adolescents’ PA using accelerometer and Global Positioning Systems is required in Hong Kong, as well as observational studies of PA during PE classes and in the schoolyard during recess, to guide the design of PA interventions.

**Supplementary Information:**

The online version contains supplementary material available at 10.1186/s12966-021-01085-z.

## Background

The health benefits of regular physical activity (PA) are well-established and include lowering risk factors for cardiovascular disease, type-2 diabetes and some cancers [1]. During childhood and adolescence, regular PA promotes favourable bone mineral density [2, 3] and is associated with reduced prevalence of risk factors for cardiovascular disease including obesity [1, 3]. There is also evidence that PA levels track across the transition from adolescence into adulthood and are, therefore, important for promoting long-term health [4]. Despite these benefits, many adolescents fail to engage in recommended levels of PA (i.e. at least one hour of moderate- to vigorous-intensity PA per day) [1]. For example, a recent pooled analysis of school-based surveys of PA among adolescents (11–17 years) in 146 countries or regions found that 81% (boys, 78%; girls, 85%) did not meet daily PA recommendations [5]. Lack of adherence to these guidelines was reported to be even worse in China (boys, 80%; girls 89%) and across the high-income Asia-Pacific region (boys, 89%; girls 96%) [5]. Adolescence is a period of decline in PA [6] and further decline may occur during the transition to adulthood [7]. Therefore, greater understanding of predictors of adolescents’ PA is needed to inform evidence-based PA interventions, especially in Asian countries which are under-represented in published research, despite Asia being the most populous continent [8].

Socio-ecological models suggest that there are multiple layers of interacting factors that may influence PA [9]. These include individual (i.e., demographic, health and psychological factors), social (e.g., social support for PA, modelling of PA behaviour), physical environmental (e.g., access to recreational facilities) and policy characteristics (e.g., urban planning policies prioritising active modes of transport). These factors may impact adolescents’ physically activity especially within the family, neighbourhood and school contexts, and evidence of their associations has been reported in several systematic reviews [10–12].

A review of studies of correlates of adolescents’ PA [10] identified several family factors that were consistently and positively associated with PA. These included support from parents and other family members, PA among siblings and opportunities for PA [10]. A more recent umbrella review of 20 systematic reviews and three meta-analyses of socio-cultural predictors of PA reported that being encouraged by family/friends to be active and having a friend, buddy or relative with whom to be active were associated with PA levels among adolescents [11]. Family socioeconomic status may be one of the determinants of family support for adolescents’ PA. For example, household income is typically associated with the provision of sports equipment, uniforms, membership fees and transport to sports opportunities [13]. Parental education is also associated with adolescents’ PA and this may be via parental modelling of PA and encouragement of adolescents’ PA (i.e., parents valuing the benefits of PA) [13]. Furthermore, household income, parental education and their perceived value of PA may influence their choice of school for their adolescent, which is described in more detail below.

The findings of a recent systematic review [12] that focused solely on Chinese youth, concurred with the finding of Sallis and colleagues [10] that parents’ PA was associated with adolescents’ PA. One distinct factor that may negatively influence PA among Chinese adolescents is the cultural influence of Confucianism, a philosophy that places less value on athletic prowess, sporting ability or success than in Western culture, and assigns greater worth to academic achievement [14]. In Chinese society, parents (especially mothers) have a strong influence on their children’s lifestyle behaviours and, among those parents who are physically active, their own engagement in PA has been shown to be consistently associated with PA among adolescents [12]. The aforementioned systematic review of predictors of PA among Chinese youth [12] recommended that further research is needed to examine explanatory environmental variables beyond the household, such as those related to school.

For all school-attending adolescents, school is a potential setting for regular PA since adolescents spend most of their waking time there on weekdays [15]. PA opportunities in the school setting are generally offered via formal physical education and recreational time at recess. In some cases, PA-friendly school policies or programs offer access to sports grounds and facilities after school or promote active transport to school [15] . Adolescents’ PA in the school context may also be influenced by social environmental factors, such as peer-support for PA [11] and physical environmental factors, such as the provision of sports equipment at recess [15]. The type of school attended can also be impactful. For example, adolescents who study at non-vocational schools are reported to be more active than those at vocational or alternative schools [13]. Despite the school being a potential setting for adolescents’ PA, the education system for primary and secondary schooling in Hong Kong is strongly focused on examinations. The learning environment is highly pressured with much time after school spent on homework and being tutored [16].

High schools in Hong Kong have a minimum requirement that 5–8% of all lesson time is allocated to physical education in junior high school (i.e. 1st–3rd years), similar to the proportions of time allocated for this in Australia (7%) [17] and the UK (8%) [18]. In senior high school (4th–6th years), the minimum allocation is 5% plus a further 5% for those students who choose physical education as an elective subject [19]. Whilst statutory regulations define the minimum time assigned to physical education classes at school, the total time overall when there are opportunities for PA (i.e., at recess and during physical education classes) is determined by school policies. Some schools may promote adolescents’ team sports as well as access to playgrounds and sports facilities after school hours and on weekends [19].

Household/family-level factors may influence adolescents’ attitude towards, and actual engagement in, PA directly and indirectly through parental choice of school. For example, adolescents who are academically-oriented may be sent to schools that focus on high achievement in examinations and entry to university, while adolescents who are more athletic or whose families provide more support for PA and/or engage in PA may choose to attend schools that allocate more time to PA, have better sports facilities and offer a greater variety of activities and sports. Family income can also influence the choice of state-run versus private schools, including privately-run ‘international’ schools, that are viewed by parents as offering a more holistic education that includes PA and sport, rather than a singular focus on academic outcomes [20].

When examining the effects of household/family- and school-level influences on adolescents’ PA, it is important to investigate individual-level psychosocial and performance characteristics as potential mediators of these associations, some of which have been identified as being consistently and positively associated with adolescents’ PA. These include (younger) age, athletic ability, attitude towards and enjoyment of PA [10] . Gender should be included as a potential correlate and moderator of associations given that adolescent boys tend to be more physically active than girls [10] and Chinese parents tend to socialise boys from an early age to be more active than girls [21]. Therefore, Chinese parents may have different expectations regarding boys’ and girls’ involvement in sports [21].

**Fig. 1:**
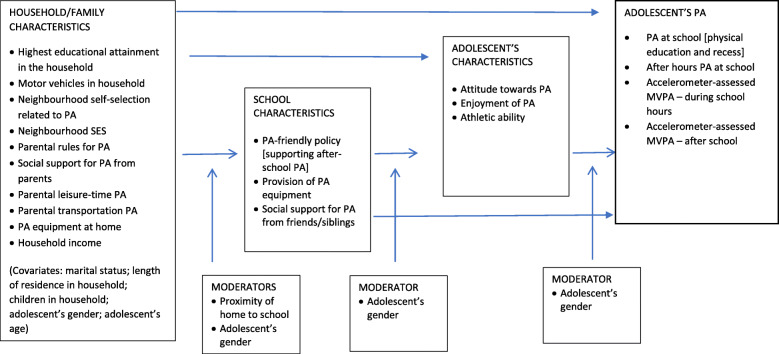
Conceptual model of household/family, school and adolescent’s characteristics, and their associations with physical activity at school

Given the lack of knowledge on factors that are associated with adolescents’ PA at school in an Asian urban setting, the aim of our study was to examine how household/family- and school-level social and physical environmental factors, and adolescents’ individual characteristics relate to adolescents’ PA in the school setting in Hong Kong, including school sports/PA programs held after school hours. The hypothesised associations are depicted in Fig. 1, which was informed by the aforementioned literature and expands on earlier studies of intrapersonal and/or family characteristics as potential correlates of PA among Chinese youth [12].

## Methods

Data were drawn from the ‘international Healthy environments and active living in teenagers – (Hong Kong)’ [iHealt(H)] study, a contributor to the ‘International Physical Activity and the Environment Network (IPEN) – Adolescent’ study that examined how the built environment is associated with adolescents’ PA and sedentary behaviour [22]. As the methods have been published previously [23], they shall be described in brief. To enable comparison of data across countries, the iHealt(H) data collection protocol aligned with that of IPEN - Adolescent project [24, 25]. Ethical approval for the iHealt(H) study was obtained from the Human Research Ethics Committee for Non-Clinical Faculties of The University of Hong Kong. Data were collected between 2013 and 15.

Adolescent and parent/carer dyads residing in pre-selected Tertiary Planning Unit (TPUs; i.e., the smallest census data collection areas in Hong Kong) stratified by census-based medium household income and transport-related walkability according to the IPEN Adolescent study protocol [24, 25] were recruited via schools. Transport-related walkability is an index of environmental factors related to walking to destinations [26]. This index comprised the sum of z-scores of residential density, street intersection density and land use mix, based on data from the Planning and Lands Departments (Hong Kong Special Administrative Region (SAR)).

Assuming a maximal cluster effect equivalent to an Intraclass Correlation Coefficient of 0.10 (based on data from our studies on adults and adolescents and studies conducted elsewhere), it was estimated that a sample of 1280 participants living in 128 TPUs would allow us to detect a 1–2% change in explained variance (small effect observed in environmental-PA studies) with 80% power under conditions of p-level of 0.05, two-tailed significance tests and > 10 covariates in a regression model. This large sample size was also needed to assess multilevel interactive effects of various factors on the outcomes.

Thirty secondary schools in different TPUs were invited to participate and twenty schools agreed to be recruited (67% response rate). To be eligible to participate, adolescent-parent dyads must have resided in their TPU for at least six months prior with the intention to stay there for the following eight months or more. The adolescent had to be aged 11–18 years and to be attending secondary school. Those who had an impairment or illness that precluded their participation in moderate-intensity PA were excluded. Classes at participating schools were randomly sampled and their adolescent students were screened for eligibility, including obtaining written parental consent and student assent for participation. In total, 2840 adolescent-parent dyads were contacted regarding the study. Some were ineligible (*n* = 738; 26%) due to non-residence in selected TPUs, or having located there within the six months prior, or having an impairment or illness that impacted on their moderate-intensity PA participation. Among the 2002 dyads who met eligibility criteria, 1363 (68% effective response rate) were recruited. Of these, 321 resided in high walkable/high income, 345 in high walkable/low income, 341 in low walkable/high income and 356 in low walkable/low income TPUs.

Within a week of receiving surveys, research staff screened all items for invalid/missing data. Participants were contacted to rectify errors or complete missing data. Invalid surveys that were unable to be corrected were received by 64 dyads who were excluded from analysis. As a result, complete data were gathered for 1299 dyads (the analytic sample).

### Measures

Paper surveys (available in English and Chinese languages) were completed by adolescents and their parent/carer at home in their own time. The parent survey included validated items and psychometric scales covering household and participating adolescent socio-demographics, parental PA, parental practices and rules related to PA, perceived neighbourhood and household environment, and reasons for living in their neighbourhood (defined as an area within 10–15 min’ walk from home [27]). The adolescent survey included validated scales translated and adapted for Hong Kong adolescents measuring PA-related psychological, social and environmental correlates [28]. A second questionnaire including all items assessing adolescents’ PA was administered to the adolescents approximately six months after the first survey to capture possible changes in activity across seasons. Psychometric properties of self-report measures of PA and correlates of PA used in the iHealt(H) study have been reported previously [28, 29].

### Outcome variables

#### Self-reported physical activity at school

This was operationalised for the current study as time available for physical activity during physical education classes and recess. Adolescents reported the frequency (days) and duration (minutes) of physical education classes per week at school, at two time-points, approximately six months apart. Similarly, they reported on the frequency and duration of recess per week. Percentage agreement for frequencies of physical education classes and recess were 98 and 94% respectively, and test-retest reliability for corresponding duration variables was good (ICC = 0.84 and 0.72, respectively). The total time per week allocated for physical education and recess were summed [28].

#### Participation in after school sport

Adolescents reported how many sports teams or ‘after school’ physical activity classes (not physical education) they participated in at school outside of standard school hours. Once again, this data was collected at two time-points, six months apart. The test re-test reliability on this item was high (ICC = 0.89; 90% agreement) [25].

#### Objectively-measured moderate-to-vigorous physical activity at school and after school

The moderate-to-vigorous PA (MVPA) of a sub-sample of adolescents (*N* = 588) was objectively measured using the ActiGraph GTX3+ accelerometer (ActiGraph LLC, Pensacola, FL) with data collected in 30 s epochs with a low frequency filter. Accelerometry data were collected at the first time-point only due to logistical issues (limited number of accelerometers). The adolescents who wore accelerometers comprised approximately 40% of the full sample and were randomly selected (stratified by TPU and gender) and were each asked to wear the waist-mounted accelerometer (placed above the right hip in a black pouch) for 7 consecutive days, for at least 10 h a day during waking hours. They also kept a wear-time diary and provided a school timetable. Non-wear time was defined as 60 min or more of consecutive zeros [30]. Inclusion criteria for data analyses were: at least 5 valid days (including 1 weekend day); valid days were considered those with 10 h of data on weekdays and 8 h of data on weekend days. Participants were asked to re-wear the accelerometer if they had an insufficient number of valid days. Of 588 adolescents, 553 had valid data (defined as above) and 35 did not (6%). In addition to criteria for the iHealth study, the current study applied further criteria of Ridgers and colleagues [31] regarding the school-related periods of interest (i.e. the school day from first to last bell, and the ‘after school’ period, operationalised here as the time between the last bell and 6 pm [32]). Objective MVPA after school hours was examined as some of this may have been school-based, however, we acknowledge this may include unstructured MVPA accrued in non-school settings. For a day to be considered valid, adolescents were required to have worn the accelerometers for ≥50% of the school day and after school period. Data were processed using ActiLife v6.0 using Evenson cut-points for PA [33].

### Exposure variables

#### Household/family characteristics

Parents reported on several socio-demographic variables including their highest educational level, choosing from the following options: (1) uneducated, (2) primary school, (3) lower secondary school, (4) higher secondary school, (5) associate degree or higher diploma, (6) Bachelor degree or (7) postgraduate degree. Further socio-demographic variables of interest were: number of motor vehicles at their household; household income per month (10 categories ranging from less than $HK 6000 to greater than $HK 59,000); marital status (categories were ‘married’, ‘widowed/divorced/separated’, ‘single and never married’ or ‘living with partner’); ethnicity/race, length of residence at current address (years, months) and number of children/adolescents in household. Neighbourhood-level socioeconomic status (SES) was determined during sample selection and based on median household income for a specific TPU.

##### Self-selection of their neighbourhood – physical activity

Parents were asked to report on seven items on self-selection of their neighbourhood that were considered likely to promote adolescents’ PA (i.e., closeness to: parks; public transport; shops and services; recreation facilities; school; presence of other youth in the neighbourhood and ease of walking) [23]. Responses were recorded on a five-point scale ranging from 1 ‘not at all important’ to 5 ‘very important’ and averaged to give a score with possible range in values of 1 to 5.

##### Parental rules for physical activity

Parents were asked whether (0 ‘no’ or 1 ‘yes’) they had each of 14 rules regarding their adolescent’s PA. These included the following: ‘Do not cross busy streets’; ‘Stay in the neighbourhood’ and ‘Do not ride bike on the street’. Responses were summed to give a score with possible range of values 0–14 [23]. This score for PA-related rules was reported previously to have good test-retest reliability (ICC = 0.75) [29].

##### Social support for physical activity from parents

Three items in the adolescent’s survey asked about parental support for PA through (1) encouragement for participation in PA or sport; (2) provision of transport to venues for PA or sport; and (3) co-participation of parent and adolescent in PA or sport. Responses were recorded on a five-point scale from 0 ‘Never’ to 4 ‘Very often’, summed and averaged to give a composite score for parental support for PA, which had good test-retest reliability (ICC = 0.78) and high internal consistency (Cronbach’s α = 0.81) [29, 34].

##### Physical activity equipment at home

Adolescents were asked to report on whether (0 ‘no’; 1 ‘yes’) they had the following ten pieces of sports equipment in or around their home: bicycle; basketball hoop; jump rope; active video games; sport equipment (e.g. balls, racquets, bats); swimming pool; rollerblades/skateboard/scooter; home aerobic equipment (e.g. treadmill, stationary bike); weight-lifting equipment; water or snow equipment (e.g. skis, kayak, snowboard). The test-retest reliability of these items, which have limited variability, has been assessed using percentage agreement which was reported as 55–67-% [35]. A score for PA equipment at home, with a test-retest ICC of 0.98, was derived by summing the responses to individual items [29].

##### Parental leisure-time and transportation physical activity

Time (minutes/week) spent in parental leisure-time PA (LTPA) and transportation PA were each measured by items specific to those domains in the Chinese version of the International Physical Activity Questionnaire (Long form) [36]. Both items have good test-retest reliability (ICC = 0.88) [36].

#### School characteristics

#### Physical activity equipment at school

Adolescents were asked to report on whether (0 ‘no’; 1 ‘yes’) they had the following six pieces of sports equipment at their school: basketball hoops; soccer goal posts; balls; running/walking track; weight machines; indoor exercise machines (e.g. treadmills). The test-retest reliability of these items, which have limited variability, has been assessed using percentage agreement which was reported as 78–86% [35]. A score for PA equipment at school was derived by summing the responses to individual items [29]. The test-retest reliability of this score quantified using ICC was 0.74 [25].

##### Physical activity-friendly policy at school

PA-friendly policy at school was measured by two items that used a five-point scale to measure the adolescent’s perception of how often (0, ‘never’ to 4 ‘always’) their school 1) offered supervised physical activities after school, and 2) allowed students to access playgrounds and sports fields after school hours [29, 35]. A composite score for PA-friendly policy at school was created by averaging responses for these two items.

##### School physical activity-friendly index

A school PA-friendly index was computed by summing the standardised scores (z scores) of PA equipment at school and PA-friendly policy at school.

##### Social support for physical activity from friends/siblings

Social support for PA from friends/siblings was measured by two items that used a five-point scale to measure the adolescent’s perception of how often (0, ‘never’ to 4 ‘very often’) friends or siblings were physically active or played sports with them, and how often they asked the adolescent to walk or cycle to school or a friend’s home. A composite score for social support for PA from friends/siblings was created by averaging responses for these two items.

#### Adolescent’s characteristics

##### Attitude towards physical activity

Attitude towards PA was measured via the adolescent’s responses to five positive and five negative statements about this. Responses were recorded on a four-point Likert scale ranging from 1 ‘strongly disagree’ to 4 ‘strongly agree’. Items included ‘Physical activity would help me stay fit’ and ‘I do not like the way physical activity and exercise makes me feel’. Response values for negative statements were reverse-coded, then all response values were averaged to give an overall score for attitude towards PA [29, 34].

##### Enjoyment of physical activity

Enjoyment of PA was measured using a single item that asked each adolescent their level of agreement with the statement, ‘I enjoy doing physical activity’. Possible responses (measured on a five-point Likert scale) ranged from 1 ‘strongly disagree’ to 5 ‘strongly agree’.

##### Athletic ability (self-rated)

Adolescents were asked to rate their athletic ability, relative to their (same gender) peers using a five-point scale, with responses ranging from 1 ‘much lower’ to 5 ‘much higher’.

### Data analytic plan and hypotheses

Descriptive statistics were computed for all variables. To estimate how household/family-level and school-level factors, and adolescents’ individual characteristics were related to adolescents’ PA in the school setting in Hong Kong, and to identify potential mediators of these associations, generalized additive mixed models (GAMMs [37];) were used. The analytical approach followed the assumptions of the model presented in Fig. 1, which hypothesised that (1) the associations of household / family characteristics with adolescents’ PA in the school setting would be partially mediated by school and adolescents’ characteristics, (2) the associations of school characteristics with adolescents’ school-based PA would be partially mediated by adolescents’ characteristics, (3) proximity of school and adolescents’ gender would moderate the associations of household / family characteristics with school characteristics, and (4) adolescents’ gender would moderate the associations of school characteristics with adolescents’ characteristics and school-based PA.

The presence of mediation effects was examined using the joint-significance test [38] and following the steps outlined in Table S1 for the full sample (Table S4 for sub-sample). According to this test, mediation is confirmed if the associations (regression coefficients) between an exposure and its mediator(s), and the exposure-adjusted associations between the mediator(s) and the outcome are statistically significant. Step 1 of the mediation analysis involved examining the total effects of each of the household / family characteristics on each of our two school characteristic outcomes (i.e., a combined measure of access to PA equipment and PA-friendly school policy; and social support for PA) (Steps 1a to 1 k in Table S1 for full sample (Table S4 for sub-sample)). Proximity of home to school and adolescent’s gender were each examined as potential moderators of associations between household / family characteristics and school characteristics. It is possible that associations between household/family characteristics and choice of school can depend on proximity to school. These associations may be stronger for schools located further from home than for those located closer to home. For example, where parents are less concerned about the sports facilities or PA opportunities offered by schools, they are more likely to choose to send their adolescent to the closest school, which may or may not be PA oriented. In contrast, some parents who value PA may consider it worth travelling a greater distance to a school if it offers a specific focus on sport [39]. Adolescent’s gender was examined as a moderator of associations between household / family characteristics and school characteristics because boys tend to engage in more physical activity than girls [6] and, from an early age, Chinese boys may be encouraged by their parents to be more physically active, when compared with girls. A study of Chinese parents of toddlers reported that mothers and fathers engaged in varying amounts and types of play with their children, according to the child’s gender [21]. Mothers tended to engage in social games with their daughters while fathers engaged in physically active play with their sons [21]. Therefore, among parents who consider PA to be important, those with adolescent boys rather than girls may place more emphasis on choosing a school that has good sports facilities.

Step 2 of the mediation analysis (Table S2, full sample; Table S5, sub-sample) entailed regressing household / family characteristics and school characteristics on adolescent’s characteristics to estimate the direct effects of school social and physical environmental variables and household characteristics on adolescent’s characteristics. Here, the moderating effects of adolescent's gender on the associations between school and adolescent's characteristics were also examined. In Step 3 (Table S3, full sample; Table S6, sub-sample) we examined the direct effects of household / family, school and adolescent's characteristics on PA outcomes at school and the moderating effects of adolescent's gender on the associations between school characteristics and school-based PA. All GAMMs were adjusted for potential confounders identified using directed acyclic graphs as detailed in the supplementary material (Additional file 1). The GAMMs explaining school-based self-reported PA outcomes also included a dichotomous time variable as a covariate and an additional random intercept variance component (within-person level) because these PA outcomes were measured twice (six months apart) in all participants. All analyses were conducted in R version 3.6.3 [40] using the packages ‘mgcv’ version 1.8.31 [41] and ‘multcomp’ version 1.4.13 [42].

## Results

Our sample comprised 1299 adolescent-parent dyads whose demographics are presented in Table 1. Most of the participating parents were mothers or female carers (76%). The mean age of parents was 44.9 (SD 6.4) years and almost all (90%) were married or in a de facto relationship. In just over a third of households, the highest education level attained was a university degree and the median number of children was 2 (IQR 1). The average length of residence at the current address was 9.7 (SD 6.6) years. Most households (69%) did not have a motorised vehicle and only 8% had two or more vehicles. Half of the sample of parents accumulated 60 min or more of transport-related PA per week, while less than half of parents engaged in any leisure-time PA. The mean age of adolescents was 14.7 (SD 1.6) years; just over half (57%) were girls, and almost all (96%) attended public schools. The median duration of self-reported PA at school at each time-point was around 2.5 h per week and half of the adolescents participated in one or more after-school sports team/class.

**Table 1:** Sample characteristics

Variables [theoretical range]	Full sample (***N*** = 1299)		Accelerometry based sub-sample of adolescents (*N* = 553)	
Household/family characteristics	*%*		*%*	
Adolescent’s gender (girls)	57.04		54.25	
Parent’s gender (female)	76.44		76.49	
	*Mean (SD)*	*Median (IQR)*	*Mean (SD)*	*Median (IQR)*
Adolescent’s age (years)	14.70 (1.57)	14.62 (2.57)	14.65 (1.56)	14.58 (2.37)
Parent’s age (years)	44.95 (6.45)	45.00 (9.00)	45.29 (6.13)	45.00 (8.00)
Length of residence at current address (years)	9.68 (6.62)	10.00 (10.00)	9.47 (6.74)	9.00 (10.08)
Number of children in the household	1.66 (0.75)	2.00 (1.00)	1.72 (0.75)	2.00 (1.00)
PA equipment at home [0–10]	4.98 (2.41)	5.00 (4.00)	4.96 (2.36)	5.00 (3.00)
Social support for PA from household adults [0–4]	1.46 (0.94)	1.33 (1.33)	1.52 (0.95)	1.33 (1.33)
Parental rules about activity [0–14]	9.24 (3.61)	10.00 (5.00)	9.51 (3.56)	10.00 (5.00)
PA-related reasons for living in the neighbourhood (neighbourhood self-selection) [1–5]	3.38 (0.74)	3.43 (1.00)	3.43 (0.74)	3.43 (1.00)
Parental transport-related PA (min/week)	166.61 (284.60)	60.00 (210.00)	165.12 (289.13)	70.00 (210.00)
Parental leisure-time PA (min/week)	122.65 (279.07)	0.00 (120.00)	128.67 (278.74)	0.00 (150.00)
Proximity to school [1–5]	2.19 (1.28)	2.00 (2.00)	2.17 (1.30)	2.00 (2.00)
Number of motorised vehicles in the household	*%*	*n*	*%*	*n*
0	69	898	68	377
1	23	298	23	128
2 or more	8	103	9	48
Monthly household income (HKD)				
< 15,000	29	380	27	152
15,000 – 29,999	30	390	30	165
30,000 – 59,999	19	248	21	116
≥ 60,000	22	281	22	120
Household highest education level				
Lower secondary or below	20	265	21	117
Higher secondary	34	438	32	179
Associate Degree/Higher diploma	12	149	11	63
Bachelor or above	34	447	35	194
Marital status (Married or in de facto relationship)	90	1163	90	498
Type of household (Apartment/unit /flat)	89	1160	87	483
Area level socio-economic status (High)	47	604	47	259
Ethnicity/race (Chinese)	90	1163	88	486
**School characteristics**	*Mean (SD)*	*Median (IQR)*	*Mean (SD)*	*Median (IQR)*
PA equipment at school [0–6]	4.53 (1.11)	5.00 (1.00)	4.58 (1.08)	5.00 (1.00)
PA-friendly policy supporting after school PA [0–4]	2.53 (0.84)	2.50 (1.00)	2.55 (0.86)	2.50 (1.00)
Social support for PA from peers [0–4]	1.15 (1.04)	1.00 (2.00)	1.26 (1.10)	1.00 (2.00)
	%	n	%	n
School type (Public)	96	1242	95	525
**Adolescent’s characteristics**	*Mean (SD)*	*Median (IQR)*	*Mean (SD)*	*Median (IQR)*
Enjoyment of PA [1–5]	3.73 (1.03)	4.00 (2.00)	3.85 (1.03)	4.00 (2.00)
Attitude towards PA [1–5]	3.17 (0.41)	3.20 (0.60)	3.20 (0.41)	3.20 (0.60)
Athletic ability [1–5]	2.84 (1.06)	3.00 (2.00)	2.92 (1.03)	3.00 (2.00)
**Adolescent’s self-reported PA**				
Total weekly minutes of PA at school				
Time 1	155.8 (55.99)	150.0 (54.00)	161.8 (58.26)	155.0 (56.00)
Time 2	163.8 (53.64)	155.0 (50.00)	168.2 (57.09)	155.0 (60.00)
Participation in after school sports teams (#) [0–4]				
Time 1	1.09 (1.17)	1.00 (2.00)	1.15 (1.21)	1.00 (2.00)
Time 2	1.02 (1.14)	1.00 (2.00)	1.08 (1.19)	1.00 (2.00)
**Accelerometry data**				
Average MVPA (minutes per day)				
At school			16.04 (9.48)	13.88 (9.78)
After school			9.59 (6.49)	8.13 (8.33)
Time spent in MVPA as % of wear time				
At school			3.70 (2.18)	3.19 (2.24)
After school			7.53 (4.94)	6.64 (6.11)
Wear time as % of all time				
At school			95.62 (6.40)	99.04 (6.90)
After school			94.04 (8.85)	99.00 (9.80)
Duration of monitoring period				
Hours of school time per day			7.58 (0.48)	7.50 (0.83)
Hours of after school time pre day			2.23 (0.30)	2.33 (0.50)
Valid days at school			4.93 (1.29)	5.00 (1.00)

SD = standard deviation; IQR = interquartile range; PA = physical activity; HKD = Hong Kong dollars; # = number

Time 1, Time 2 – data were collected at two time-points six months apart

Our sub-sample comprised 553 adolescent-parent dyads, where the adolescent had worn an accelerometer. Overall, there was very little difference in the values of most demographic variables among the sub-sample, compared with the full sample (Table 1). However, the median time spent in transport-related PA was ten minutes higher among the parents of the sub-sample, compared with the full sample. As for the whole sample, the median duration of self-reported PA at school at each time-point was around 2.5 h per week. Half of the adolescents accrued around 70 min or more per week (almost 14 min per school day) of MVPA at school and at least 40 min of MVPA per week (over 8 min per school day) after school hours. On average, boys accrued significantly more MVPA than girls during school hours (boys, mean 19.6 (SD 10.1) minutes per day; girls, mean 13.0 (SD 7.8) minutes per day; *p* < 0.001) and after school (boys, mean 10.4 (SD 7.1) minutes per day; girls, mean 8.9 (SD 5.8) minutes per day; *p* = 0.006; data not shown in Table 1).

### Correlates of self-reported physical activity (full sample)

**Fig. 2:**
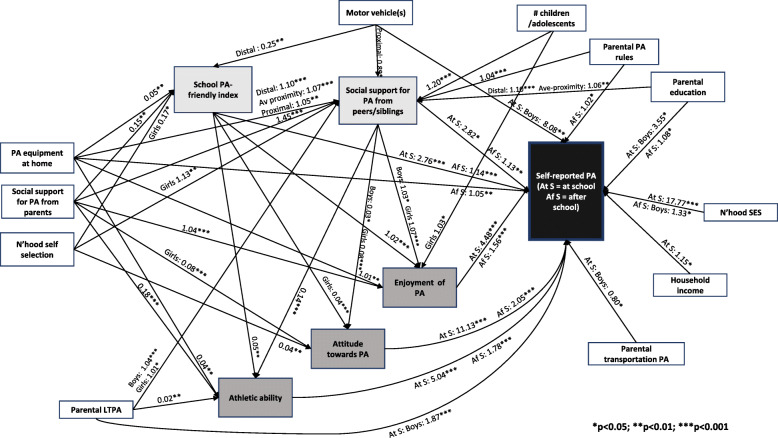
Direct and indirect effects of household/family, school and adolescent’s characteristics on self-reported school-based physical activity. Regression coefficients for social support for PA from peers/siblings, enjoyment of PA and self-reported school-based PA after school represent exponentiated values

The direct and indirect pathways between household/family, school and adolescent’s characteristics, and adolescent’s self-reported PA are depicted in Fig. 2. Social support for PA from parents showed positive indirect effects on self-reported PA at school during and after school hours (Fig. 2). This is because social support for PA from parents was positively associated with all school and adolescent’s characteristics (Tables 2 and 3), and the latter were positively related to self-reported PA at school during and after school hours (Table 4). Also, PA-friendly school characteristics were positively associated with enjoyment of PA and athletic ability in all adolescents, and with attitude towards PA mainly in girls (Table 3). A similar pattern of indirect effects on self-reported PA was also observed for PA equipment at home (Fig. 2; Tables 2, 3 and 4), with the exception that no significant association between this household characteristic and adolescent's attitude towards PA was observed (Table 3). In addition, we found a positive direct effect of PA equipment at home and self-reported PA at school after school hours (Fig. 2; Table 4).

**Table 2:** Total effects of household/family characteristics on school characteristics and the moderating effects of proximity to school and adolescent’s gender (Step 1 of mediation analysis)

Household / family characteristics	Effect	School PA-friendly index^**‡**^		Social support for PA from peers	
		*b* (95% CI)	p	e^*b*^ (95% CI)	p
Highest educational attainment in the household	Main - total	0.06 (−0.01, 0.12)	0.082	**1.05 (1.02, 1.09)**	**0.005**
	@ below-average proximity of home to school	N/A	N/A	**1.10 (1.05,1.16)**	**< 0.001**
	@ average proximity of home to school	N/A	N/A	**1.06 (1.02,1.10)**	**0.003**
	@ above-average proximity of home to school	N/A	N/A	1.02 (0.96,1.08)	0.479
Number of motor vehicles in the household	Main	**0.14 (0.02,0.26)**	**0.027**	0.97 (0.90,1.04)	0.369
	@ below-average proximity of home to school	**0.25 (0.10,0.40)**	**0.001**	1.03 (0.94,1.12)	0.588
	@ average proximity of home to school	0.11 (−0.01,0.23)	0.069	0.95 (0.88,1.03)	0.225
	@ above-average proximity of home to school	−0.03 (− 0.21,0.15)	0.738	**0.89 (0.79,1.00)**	**0.044**
Number of children in the household	Main - total	0.05 (−0.06,0.16)	0.393	**1.20 (1.12,1.28)**	**< 0.001**
Neighbourhood self-selection related to PA	Main - total	0.07 (−0.04,0.18)	0.193	1.06 (0.99,1.13)	0.116
	In boys	−0.04 (− 0.20,0.11)	0.595	0.97 (0.88,1.07)	0.561
	In girls	**0.17 (0.02,0.31)**	**0.023**	**1.13 (1.04,1.24)**	**0.007**
Neighbourhood socio-economic status	Main - total	0.04 (−0.22,0.29)	0.777	1.12 (0.99,1.26)	0.079
Household income	Main - total	−0.02 (− 0.05,0.02)	0.437	1.00 (0. 97,1.02)	0.816
Parental rules for PA	Main - total	0.01 (−0.01,0.03)	0.311	**1.04 (1.02,1.05)**	**< 0.001**
Social support for PA from parents	Main - total	**0.15 (0.06,0.25)**	**0.001**	**1.45 (1.37,1.54)**	**< 0.001**
PA equipment at home / neighbourhood	Main - total	**0.05 (0.01,0.08)**	**0.007**	**1.07 (1.05,1.09)**	**< 0.001**
	@ below-average proximity of home to school	N/A	N/A	**1.10 (1.06,1.13)**	**< 0.001**
	@ average proximity of home to school	N/A	N/A	**1.07 (1.05,1.09)**	**< 0.001**
	@ above-average proximity of home to school	N/A	N/A	**1.05 (1.02,1.08)**	**0.002**
Parental leisure-time PA^	Main - total	0.01 (−0.00,0.03)	0.133	**1.02 (1.01,1.03)**	**< 0.001**
	In boys	N/A	N/A	**1.04 (1.02,1.06)**	**< 0.001**
	In girls	N/A	N/A	**1.01 (1.00,1.03)**	**0.021**
Parental transportation PA^	Main - total	0.00 (−0.01,0.02)	0.726	1.01 (0.99,1.02)	0.274

PA = physical activity; ^‡^ consisting of items measuring PA equipment at school and school PA-friendly policy supporting after-school PA

*b* = regression coefficient; CI = confidence interval; e^*b*^ = exponentiated regression coefficient; N/A denotes ‘no significant interaction’; ^ minutes converted to hours (by dividing by 60). These regression models were adjusted for confounders listed in Table S1

**Table 3:** Effects of household/family characteristics and school characteristics on adolescent’s characteristics and the moderating effects of adolescent’s gender (Step 2 of mediation analysis)

Household / family characteristics	Effect	Attitude towards PA		Enjoyment of PA		Athletic ability	
		*b* (95% CI)	p	e^*b*^ (95% CI)	p	*b* (95% CI)	p
Highest educational attainment in household (main effect)	Main - direct	−0.01 (− 0.03,0.01)	0.201	0.99 (0.98,1.00)	0.203	0.00 (−0.04,0.05)	0.888
No. of motor vehicles in household	Main - direct	−0.01 (− 0.05,0.02)	0.412	0.99 (0.96,1.01)	0.201	−0.01(− 0.09,0.08)	0.901
No. of children in household	Main - direct In boys In girls	0.01 (−0.02,0.04) N/A N/A	0.410 N/A N/A	1.00 (0.98,1.02) 0.97 (0.94,1.01) **1.03 (1.00,1.06)**	0.864 0.101 **0.022**	0.01 (−0.06,0.09) N/A N/A	0.739 N/A N/A
Neighbourhood self-selection for PA	Main - direct	**0.04 (0.01,0.07)**	**0.007**	1.02 (1.00,1.04)	0.088	−0.03(− 0.10,0.05)	0.490
Neighbourhood socio-economic status	Main - direct	0.01 (−0.05,0.07)	0.685	1.02 (0.98,1.05)	0.337	−0.01(− 0.13,0.11)	0.875
Household income	Main - direct	0.01 (−0.00,0.02)	0.085	1.00 (0.99,1.01)	0.713	0.00 (−0.02,0.03)	0.752
Parental rules for PA	Main - direct	0.00 (−0.01,0.01)	0.956	1.00 (0.99,1.00)	0.421	−0.00(− 0.02,0.01)	0.396
Social support for PA from parents	Main - direct	**0.06 (0.03,0.09)**	**< 0.001**	**1.04 (1.02,1.06)**	**< 0.001**	**0.18 (0.11,0.25)**	**< 0.001**
	In boys	0.03 (−0.01,0.07)	0.102	N/A	N/A	N/A	N/A
	In girls	**0.08 (0.05,0.11)**	**< 0.001**	N/A	N/A	N/A	N/A
PA equipment at home/neighbourhood	Main - direct	0.00 (−0.01,0.01)	0.896	**1.01 (1.00,1.02)**	**0.001**	**0.04 (0.01,0.06)**	**0.003**
Parental leisure-time PA^	Main - direct	0.00 (−0.00,0.01)	0.115	1.00 (1.00,1.01)	0.293	**0.02 (0.01,0.03)**	**0.002**
Parental transportation PA^	Main - direct	−0.00 (− 0.01,0.00)	0.381	1.00 (0.99,1.0)	0.458	0.00 (−0.01,0.01)	0.878
School PA-friendly index^‡^	Main - total	**0.03 (0.01,0.04)**	**< 0.001**	**1.02 (1.01,1.03)**	**< 0.001**	**0.06 (0.02,0.09)**	**0.003**
	Main - direct	**0.03 (0.01,0.04)**	**< 0.001**	**1.02 (1.01,1.03)**	**< 0.001**	**0.05 (0.01,0.09)**	**0.005**
	In boys	0.01 (−0.01,0.03)	0.499	N/A	N/A	N/A	N/A
	In girls	**0.04 (0.02,0.06)**	**< 0.001**	N/A	N/A	N/A	N/A
Social support from peers	Main - direct	**0.06 (0.04,0.08)**	**< 0.001**	**1.05 (1.03,1.06)**	**< 0.001**	**0.14 (0.08,0.20)**	**< 0.001**
	In boys	**0.03 (0.00,0.06)**	**0.036**	**1.03 (1.01,1.05)**	**0.010**	N/A	N/A
	In girls	**0.08 (0.05,0.11)**	**< 0.001**	**1.07 (1.05,1.09)**	**< 0.001**	N/A	N/A

PA = physical activity; *b* = regression coefficient; CI = confidence interval; e^*b*^ = exponentiated regression coefficient; N/A denotes ‘no significant interaction’

^ minutes converted to hours (by dividing by 60); ^‡^ consisting of items measuring PA equipment at school and school PA-friendly policy supporting after-school PA. These regression models were adjusted for confounders listed in Table S2

**Table 4:** Effects of household/family characteristics, school characteristics and adolescent’s characteristics on adolescent’s physical activity at school, and moderating effects of adolescent’s gender (Step 3 of mediation analysis)

Household / family characteristics	Effect	PA at school during school hours		PA at school after school hours	
		*b* (95% CI)	p	e^*b*^ (95% CI)	p
Highest educational attainment in household	Main - direct In boys In girls	1.57 (−0.48,3.62) **3.55 (0.83, 6.26)** 0.11 (−2.38,2.60)	0.134 **0.010** 0.931	**1.08 (1.01,1.15)** N/A N/A	**0.027** N/A N/A
No. of motor vehicles in household	Main - direct	**4.08 (0.63,7.54)**	**0.021**	1.11 (1.00,1.24)	0.054
	In boys	**8.08 (2.72,13.44)**	**0.003**	N/A	N/A
	In girls	1.79 (− 2.36,5.94)	0.398	N/A	N/A
No. of children in household	Main - direct	2.24 (−0.91,5.39)	0.164	1.09 (0.97,1.22)	0.164
Neighbourhood self-selection for PA	Main -direct	−1.76 (−4.83,1.31)	0.262	0.94 (0.84,1.04)	0.202
Neighbourhood socio-economic status	Main - direct	**17.77 (9.27,26.27)**	**< 0.001**	1.11 (0.91,1.37)	0.305
	In boys	N/A	N/A	**1.33 (1.02,1.74)**	**0.035**
	In girls	N/A	N/A	0.97 (0.76,1.23)	0.781
Household income	Main - direct	**1.15 (0.06,2.24)**	**0.039**	0.99 (0.96,1.03)	0.601
Parental rules for PA	Main - direct	0.41 (−0.23,1.05)	0.206	**1.02 (1.00,1.04)**	**0.040**
Social support for PA from parents	Main - direct	1.04 (−1.84,3.91)	0.479	1.05 (0.96,1.15)	0.308
	In boys	3.86 (− 0.03,7.74)	0.052	N/A	N/A
	In girls	−1.17 (−4.76,2.42)	0.523	N/A	N/A
PA equipment at home / neighbourhood	Main - direct	0.26 (−0.72,1.23)	0.607	**1.05 (1.02,.1.09)**	**0.003**
Parental leisure-time PA^	Main - direct	**0.62 (0.12,1.12)**	**0.016**	1.01 (1.00,1.03)	0.083
	In boys	**1.87 (0.93,2.81)**	**< 0.001**	N/A	N/A
	In girls	0.23 (− 0.35,0.81)	0.438	N/A	N/A
Parental transportation PA^	Main - direct	0.22 (−0.24,0.69)	0.351	1.01 (0.99,1.02)	0.377
	In boys	**0.80 (0.11,1.49)**	**0.023**	N/A	N/A
	In girls	−0.27 (− 0.89,0.35)	0.392	N/A	N/A
**School Characteristics**					
School PA-friendly index^‡^	Main - direct	**2.76 (1.19,4.33)**	**< 0.001**	**1.14 (1.08,1.20)**	**< 0.001**
Social support from peers	Main - direct	**2.82 (0.43,5.22)**	**0.021**	**1.13 (1.04,1.22)**	**0.003**
**Adolescent’s characteristics**					
Attitude towards PA	Main - direct	**11.13 (5.36,16.91)**	**< 0.001**	**2.05 (1.66,2.52)**	**< 0.001**
Enjoyment of PA	Main - direct	**4.48 (2.21, 6.75)**	**< 0.001**	**1.56 (1.43,1.69)**	**< 0.001**
Athletic ability	Main - direct	**5.04 (2.84,7.24)**	**< 0.001**	**1.78 (1.65,1.92)**	**< 0.001**

PA = physical activity; *b* = regression coefficient; CI = confidence interval; e^*b*^ = exponentiated regression coefficient; N/A denotes ‘no significant interaction’

^ minutes converted to hours (by dividing by 60); ^‡^ consisting of items measuring PA equipment at school and school PA-friendly policy supporting after-school PA. These regression models were adjusted for confounders listed in Table S3

Parental education, rules regarding PA and parental LTPA all had indirect effects on both self-reported PA at school and after school via pathways linking these variables with social support for PA from peers/siblings at school (Fig. 2; Table 2), which, as described above, was positively associated with all adolescent’s characteristics (Fig. 2; Table 3), which in turn were positively related to self-reported PA at school and after school hours (Fig. 2; Table 4). Parental education, rules regarding PA and parental LTPA were also linked with both self-reported PA outcomes through direct pathways between social support from peers/siblings at school and these outcome variables (Fig. 2; Table 4). The effect of parental education on social support for PA from peers/siblings was stronger with greater distance between home and school, while the effect of parental LTPA on social support for PA from peers/siblings was stronger for boys than for girls (Table 2). Parental education also had direct effects on PA during school hours (boys only) and after school, while parental rules for PA had a direct effect only on PA after school (Table 4). Parental LTPA had a direct effect on PA during school hours, with a positive association observed only in boys (Table 4).

The number of motor vehicles per household had a direct effect on self-reported PA during school hours, particularly for boys (Table 4) plus indirect effects on both school PA outcomes via pathways through the school PA-friendly index (Tables 2 and 3), which was more strongly positively associated with number of vehicles as distance to school increased (Table 2).

Indirect effects were observed between neighbourhood self-selection for PA, number of children/adolescents in the household and self-reported PA at school (Fig. 2). The former household/family characteristic exerted an effect on PA at school via both school characteristics for girls only (Table 2), and also via adolescent’s attitude towards PA (Tables 3 and 4), while the latter characteristic followed the pathways linking social support for PA from peers/siblings with self-reported PA at school, as well as a more direct pathway via enjoyment of PA among girls only (Tables 2, 3 and 4). Household income and neighbourhood SES showed a positive direct effect but no indirect effects on self-reported PA at school during school hours (Fig. 2; Table 4). In addition, neighbourhood SES displayed a direct positive effect on self-reported PA at school after school hours but only in boys.

### Objectively-measured physical activity (sub-sample)

**Fig. 3:**
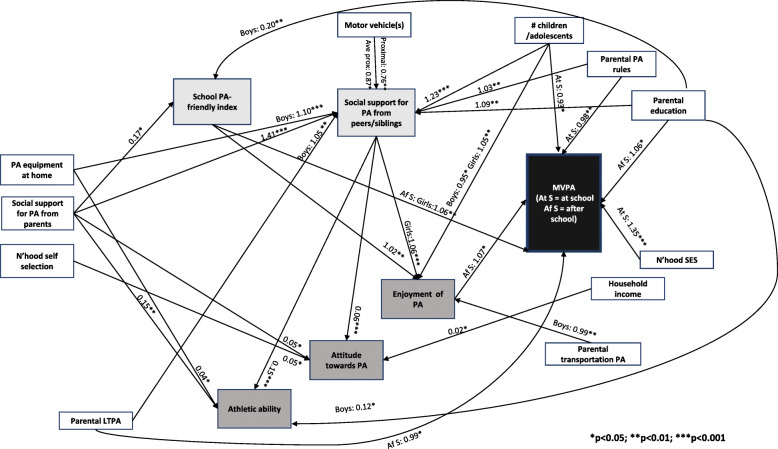
Direct and indirect effects of household/family, school and adolescent’s characteristics on objectively-measured physical activity. Regression coefficients for social support for PA from peers/siblings, enjoyment of PA and MVPA represent exponentiated values

The direct and indirect pathways between household/family, school and adolescent’s characteristics, and adolescent’s objectively-measured MVPA are depicted in Fig. 3. Social support for PA from parents showed positive indirect effects on MVPA after school hours (Fig. 3, Tables 5, 6 and 7). This is because social support for PA from parents was positively associated with both school characteristics (Table 5), which were each positively related to adolescent’s enjoyment of PA (Table 6) that, in turn, was predictive of more after-school MVPA (Table 7). The intermediate effect of social support for PA from peers/siblings on adolescent’s enjoyment of PA was stronger for girls than for boys (Table 6). Adolescent’s enjoyment of PA was positively associated with MVPA after school hours (Table 7) and was the only adolescent characteristic to do so. In addition, the school-friendly PA index had a direct effect on MVPA after school hours among girls only (Table 7). A similar pattern of indirect effects on MVPA after school hours was observed for parental education, although the pathway via the school PA-friendly index was significant for boys only (Fig. 3; Tables 5-7).

**Table 5:** Total effects of household/family characteristics on school characteristics and the moderating effects of proximity to school and adolescent’s gender for sub-sample^a^ (Step 1 of mediation analysis)

Household / family characteristics	Effect	School PA-friendly index^**‡**^		Social support for PA from peers	
		*b* (95% CI)	p	e^*b*^ (95% CI)	p
Highest educational attainment in the	Main - total	0.09 (− 0.00,0.19)	0.051	**1.09 (1.03,1.15)**	**0.002**
household	In boys	**0.20 (0.06,0.33)**	**0.004**	N/A	N/A
	In girls	0.02 (−0.10,0.13)	0.777	N/A	N/A
Number of motor vehicles in the household	Main - total	0.11 (−0.06,0.28)	0.212	0.91 (0.82,1.01)	0.090
	@ below-average proximity of home to school	N/A	N/A	1.00 (0.88,1.14)	0.966
	@ average proximity of home to school	N/A	N/A	**0.87 (0.78,0.98)**	**0.021**
	@ above-average proximity of home to school	N/A	N/A	**0.76 (0.63,0.92)**	**0.004**
Number of children in the household	Main - total	0.01 (−0.16,0.18)	0.909	**1.23 (1.11,1.36)**	**< 0.001**
Neighbourhood self-selection related to PA	Main - total	−0.04 (− 0.21,0.12)	0.607	1.01 (0.91,1.12)	0.877
	In boys	N/A	N/A	0.91 (0.78,1.05)	0.186
	In girls	N/A	N/A	1.11 (0.97,1.28)	0.127
Neighbourhood socio-economic status	Main - total	0.19 (−0.17,0.55)	0.306	1.16 (0.96,1.41)	0.128
Household income	Main - total	−0.03 (− 0.09,0.03)	0.282	0.98 (0.95,1.02)	0.371
Parental rules for PA	Main - total	−0.02 (− 0.05,0.01)	0.262	**1.03 (1.01,1.05)**	**0.007**
Social support for PA from parents	Main - total	**0.17 (0.04,0.31)**	**0.013**	**1.41 (1.30,1.53)**	**< 0.001**
PA equipment at home / neighbourhood	Main - total	0.05 (−0.01,0.10)	0.079	**1.06 (1.02,1.09)**	**< 0.001**
	In boys	N/A	N/A	**1.10 (1.05,1.15)**	**< 0.001**
	In girls	N/A	N/A	1.02 (0.97,1.06)	0.430
Parental leisure-time PA^	Main - total	0.00 (−0.03,0.03)	0.998	**1.02 (1.01,1.04)**	**0.007**
	In boys	N/A	N/A	**1.05 (1.01,1.08)**	**0.003**
	In girls	N/A	N/A	1.01 (0.99,1.03)	0.227
Parental transportation PA^	Main - total	−0.01 (−0.03,0.02)	0.531	1.00 (0.99,1.02)	0.704
	@ below-average proximity of home to school	N/A	N/A	0.99 (0.97,1.01)	0.339
	@ average proximity of home to school	N/A	N/A	1.01 (0.99,1.02)	0.476
	@ above-average proximity of home to school	N/A	N/A	1.02 (1.00,1.04)	0.058

^a^ sub-sample of adolescents who wore accelerometer; PA = physical activity; ^‡^ consisting of items measuring PA equipment at school and school PA-friendly policy supporting after-school PA; *b* = regression coefficient; CI = confidence interval; e^*b*^ = exponentiated regression coefficient; N/A denotes ‘no significant interaction’. ^ minutes converted to hours (by dividing by 60). These regression models were adjusted for confounders listed in Table S4

**Table 6:** Effects of household/family characteristics and school characteristics on adolescent’s characteristics and the moderating effects of adolescent’s gender for sub-sample^a^ (Step 2 of mediation analysis)

Household / family characteristics	Effect	Attitude towards PA		Enjoyment of PA		Athletic ability	
		*b* (95% CI)	p	e^*b*^ (95% CI)	p	*b* (95% CI)	p
Highest educational attainment in household	Main - direct	−0.00 (−0.03,0.03)	0.891	1.00 (0.98,1.02)	0.840	0.06 (−0.02,0.13)	0.133
	In boys	N/A	N/A	N/A	N/A	**0.12 (0.02,0.21)**	**0.017**
	In girls	N/A	N/A	N/A	N/A	0.01 (−0.08,0.10)	0.858
No. of motor vehicles in household	Main - direct	−0.02 (− 0.07,0.03)	0.382	0.99 (0.96,1.03)	0.678	−0.04 (− 0.16,0.08)	0.536
No. of children in household	Main - direct	0.01 (−0.04,0.05)	0.761	1.00 (0.97,1.03)	0.972	0.02 (−0.09,0.14)	0.703
	In boys	−0.04 (− 0.10,0.03)	0.247	**0.95 (0.91,0.99)**	**0.017**	N/A	N/A
	In girls	0.05 (−0.01,0.11)	0.086	**1.05 (1.01,1.10)**	**0.009**	N/A	N/A
Neighbourhood self-selection for PA	Main - direct	**0.05 (0.01,0.10)**	**0.028**	1.00 (0.97,1.03)	0.998	−0.05(−0.17,0.06)	0.381
Neighbourhood socio-economic status	Main - direct	−0.01 (− 0.11,0.09)	0.813	0.99 (0.94,1.05)	0.779	−0.01(− 0.21,0.19)	0.897
Household income	Main - direct	**0.02 (0.00,0.03)**	**0.030**	1.00 (0.99,1.01)	0.488	0.01 (−0.03,0.05)	0.674
Parental rules for PA	Main - direct	0.00 (−0.01,0.01)	0.873	1.00 (0.99,1.01)	0.652	−0.01(− 0.03,0.02)	0.463
	In boys	0.01 (−0.00,0.03)	0.065	N/A	N/A	N/A	N/A
	In girls	−0.01 (−0.02,0.01)	0.249	N/A	N/A	N/A	N/A
Social support for PA from parents	Main - direct	**0.05 (0.01,0.09)**	**0.021**	1.02 (0.99,1.05)	0.174	**0.15 (0.04,0.25)**	**0.006**
PA equipment at home/neighbourhood	Main - direct	−0.00 (−0.02,0.01)	0.718	1.01 (1.00,1.02)	0.119	**0.04 (0.00,0.08)**	**0.032**
Parental leisure-time PA^	Main - direct	0.00 (−0.00,0.01)	0.279	1.00 (1.00,1.01)	0.101	0.01 (−0.01,0.03)	0.222
Parental transportation PA^	Main - direct	−0.01 (− 0.01,0.00)	0.141	1.00 (0.99,1.00)	0.569	0.01 (−0.01,0.02)	0.507
	In boys	N/A	N/A	**0.99 (0.98,1.00)**	**0.004**	N/A	N/A
	In girls	N/A	N/A	1.01 (1.00,1.013)	0.060	N/A	N/A
School PA-friendly index^‡^	Main - total	0.01 (−0.01,0.03)	0.337	**1.02 (1.01,1.04)**	**0.003**	0.01 (−0.05,0.07)	0.707
	Main - direct	0.01 (−0.01,0.03)	0.472	**1.02 (1.01,1.04)**	**0.006**	0.01 (−0.05,0.06)	0.842
Social support from peers	Main - direct	**0.06 (0.03,0.10)**	**< 0.001**	**1.04 (1.02,1.06)**	**< 0.001**	**0.15 (0.06,0.23)**	**< 0.001**
	In boys	N/A	N/A	1.02 (1.00,1.05)	0.096	N/A	N/A
	In girls	N/A	N/A	**1.06 (1.03,1.10)**	**< 0.001**	N/A	N/A

^a^ sub-sample of adolescents who wore accelerometer; PA = physical activity; *b* = regression coefficient; CI = confidence interval; e^*b*^ = exponentiated regression coefficient. N/A denotes ‘no significant interaction’; ^ minutes converted to hours (by dividing by 60); ^‡^ consisting of items measuring PA equipment at school and school PA-friendly policy supporting after-school PA. These regression models were adjusted for confounders listed in Table S5

**Table 7:** Effects of household / family characteristics, school characteristics and adolescent’s characteristics on adolescent’s moderate-to-vigorous physical activity at school (sub-sample^a^), and the moderating effects of adolescent’s gender (Step 3 of mediation analysis)

Household / family characteristics	Effect	Average MVPA (min/day) at school during school hours		Average MVPA (min/day) after school hours	
		e^b^ (95% CI)	p	e^b^ (95% CI)	p
Highest educational attainment in the household	Main - direct	1.02 (0.98,1.06)	0.399	**1.06 (1.01,1.11)**	**0.029**
Number of motor vehicles in the household	Main - direct	0.99 (0.93,1.06)	0.816	0.99 (0.92,1.07)	0.849
Number of children in the household	Main - direct	**0.93 (0.87,0.99)**	**0.018**	0.99 (0.93,1.07)	0.891
Neighbourhood self-selection related to PA	Main -direct	0.98 (0.92,1.04)	0.462	1.02 (0.94,1.10)	0.642
Neighbourhood socio-economic status	Main - direct	**1.35 (1.18,1.54)**	**< 0.001**	1.11 (0.97,1.26)	0.124
Household income	Main - direct	0.99 (0.97,1.01)	0.337	0.99 (0.96,1.01)	0.309
Parental rules for PA	Main - direct	**0.98 (0.97,0.99)**	**0.005**	0.99 (0.97,1.00)	0.084
Social support for PA from parents	Main - direct	0.99 (0.93,1.04)	0.605	1.03 (0.96,1.10)	0.399
PA equipment at home / neighbourhood	Main - direct	0.99 (0.97,1.01)	0.542	0.99 (0.97,1.01)	0.396
Parental leisure-time PA^	Main - direct	1.00 (0.99,1.01)	0.655	**0.99 (0.98,1.00)**	**0.025**
Parental transportation PA^	Main - direct	1.00 (0.99,1.01)	0.735	1.01 (0.99,1.02)	0.275
**School Characteristics**					
School PA-friendly index^‡^	Main - direct	0.99 (0.96,1.02)	0.462	1.02 (0.98,1.06)	0.285
	In boys	N/A	N/A	0.98 (0.93,1.03)	0.455
	In girls	N/A	N/A	**1.06 (1.02,1.12)**	**0.008**
Social support from peers	Main - direct	1.00 (0.95,1.04)	0.884	0.97 (0.92,1.02)	0.287
**Adolescent’s characteristics**					
Attitude towards PA	Main - direct	1.13 (0.99,1.29)	0.073	1.09 (0.93,1.28)	0.275
Enjoyment of PA	Main - direct	1.03 (0.97,1.08)	0.332	**1.07 (1.01,1.15)**	**0.032**
Athletic ability	Main - direct	1.04 (0.99,1.09)	0.104	1.03 (0.97,1.09)	0.373

^a^ sub-sample of adolescents who wore accelerometer; MVPA = moderate to vigorous physical activity; e^*b*^ = exponentiated regression coefficient

CI = confidence interval; PA = physical activity; ^ minutes converted to hours (by dividing by 60); ^‡^ consisting of items measuring PA equipment at school and school PA-friendly policy supporting after-school PA; N/A denotes ‘no significant interaction’. These regression models were adjusted for confounders listed in Table S6

Several other household/family characteristics showed positive indirect effects on MVPA after school hours via intermediate pathways through only one school characteristic, social support for PA from peers/siblings, and adolescent’s enjoyment of PA. The intermediate pathway between the latter two variables was significant mainly for girls (Table 6). These were: number of motor vehicles, number of children/adolescents in household and parental rules regarding PA (Fig. 3; Tables 5, 6 and 7). Pathways from PA equipment at home and parental LTPA via social support for PA from peers/siblings were limited since the initial association with this school variable was mainly for boys (and the intermediate association between social support for PA from peers/siblings and enjoyment of PA was mainly for girls). There was also a more direct pathway between number of children/adolescents in household and MVPA after school hours via adolescent’s enjoyment of PA, which was positive for girls but negative for boys (Table 6). The effect of the number of motor vehicles on social support for PA from peers/siblings was stronger with school being closer to home (Table 5).

Direct effects on MVPA at school were observed for number of children/adolescents in the household, neighbourhood socioeconomic status and parental rules regarding PA (Fig. 3; Table 7). In addition, direct effects on MVPA after school hours were observed for parental education and parental LTPA (Fig. 3; Table 7).

## Discussion

This study aimed to examine the impact of household/family-, school- and individual characteristics on PA at and after school, a setting in which adolescents spend almost half of their waking time on weekdays. Our study differs from previous studies of adolescents’ PA at school (and more broadly) [15, 43, 44] because it does not just examine independent direct effects of household/family variables, school variables and adolescent variables on school-based PA. Instead, we adopted an approach that allows investigation of pathways through which these variables impact on PA via other more proximal variables (e.g. individual adolescent’s characteristics). This approach increases the power to detect distal influences that may otherwise go undetected [45]. As a result, a complex network of potential pathways of influence on adolescents’ school-based PA was identified. Overall, most of the significant effects were indirect ones. However, there were far fewer significant pathways between household/family characteristics and objectively-measured MVPA at school than there were for self-reported PA at school. In fact, there were no indirect pathways between these variables and MVPA at school.

Before discussing the identified pathways in more detail, several plausible explanations are suggested for these disparities in associations for self-reported PA compared with objectively-measured PA. Previous studies suggest that adolescents may over-estimate their PA using self-report measures and/or may have difficulty with recall [46, 47]. Our self-reported measure of PA at school measured time available for opportunities to be physically active, i.e. during PE classes and recess. However, PE classes may include time spent being sedentary, such as during instruction or taking a turn ‘on the bench’ during team sports [15, 48]. A systematic review of studies of time spent in MVPA during PE classes at high school found that just over a third (36%) of class-time was spent in MVPA, with management, instruction and motivation of students accounting for much of the remaining time [49]. Further, adolescents are not necessarily active during recess time since time may be spent eating snacks, using toilet facilities, or engaging in sedentary behaviours. Girls may view recess as a social opportunity for sitting and chatting with friends [15]. Another potential explanation is that the sub-sample of adolescents who wore accelerometers may be underpowered to detect moderation by gender or distance to school. However, given that our sub-sample still contained objective PA data for over 500 adolescents, our findings could indicate that the expectations of parents/adolescents regarding engagement in PA were not being met. As such, this may have policy implications in that schools may need to re-design PE lessons so that students engage in greater levels of PA than observed in this study [49].

Our discussion of pathways shall begin with the most proximal associations (e.g., from adolescent’s characteristics to PA outcomes) as they are simpler to explain and then work backwards to those that are more complex. Adolescent’s attitude towards PA, enjoyment of PA and athletic ability were all positively associated with both self-reported PA variables. However, only enjoyment of PA was associated with objectively-measured MVPA, and this was only for after school hours. This is not surprising as enjoyment of PA is related to intrinsic motivation to be physically active [50] and has been identified as a significant predictor of PA among children and adolescents [23, 51, 52]. A similar construct, enjoyment of PE, has been shown to be associated with participation in sports teams [53], which was included in our measure of self-reported PA after school.

Among the full sample, both school characteristics were positively associated with all adolescent characteristics, as well as being directly associated with self-reported PA at school and after school. All household/family characteristics except neighbourhood SES and parental transportation PA had indirect effects on both self-reported school PA outcome variables via the pathways described above through one or both school characteristics. Among the sub-sample of adolescents who wore accelerometers, the school PA-friendly index was associated directly with MVPA after school, only among girls, and indirectly with MVPA after school among boys and girls via adolescent’s enjoyment of PA. Among girls, social support for PA from peers/siblings was indirectly associated with MVPA after school via enjoyment of PA. Through their association with social support for PA from peers/siblings, parental education, numbers of motor vehicles and children/adolescents in the household, as well as parental rules for PA, were all associated indirectly with MVPA after school mainly for girls. It is not surprising that social support for PA from peers/siblings appears to be more important for girls than for boys. Differences in PA levels at school among adolescent girls compared with boys in our study reflect findings globally that girls are less physically active than boys at school [15, 54] and overall [1, 55], and that adolescence is a key life stage when girls exhibit low PA levels [6, 55]. However, peer support and PA options that offer social interaction have been identified as important facilitators for PA among adolescent girls [56].

Social support for PA from parents had indirect pathways to MVPA after school for boys and girls, via the school PA-friendly index with and without enjoyment of PA. It is possible that parents who value PA and provide social support for their offspring’s PA more broadly may tend to choose a school that has PA-friendly policy and facilities [57]. Evidently, there is scope for choice considering data from a study of almost half (45%) of schools in Hong Kong that demonstrate broad diversity in PA-friendly policy, PA ethos, PA equipment and playground size [57]. A further direct but inverse association between parental transportation PA and enjoyment of PA among adolescent boys may be due to this being associated with their own active transport [23]. If boys engage in PA via walking or cycling by necessity (e.g., lack of a motor car) rather than by desire or choice, they may not perceive this to be enjoyable.

The direct associations of several factors with self-reported PA at school were significant either in boys only (parental education; parental transportation PA) or mainly in boys (number of motor vehicles in household; parental LTPA). In addition, the direct association of neighbourhood SES with self-reported PA after school was significant for boys only. It is possible that more educated (and affluent) parents value the health benefits of PA, evidenced by their own LTPA and transportation PA and choose schools for their children that offer greater opportunities for school-based PA. However, the observation of these associations among boys, in particular, may reflect the traditional, patriarchal values of Confucianism that promote strong, active behaviour among males, in contrast to weak, passive behaviour among females. This may impact girls’ PA since, in Confucianism, women are forbidden to talk about physique or dress in a way that exposes their form (e.g., by wearing Western-style exercise clothing) [58].

There were few direct associations of household/family characteristics with objectively-measured MVPA. The number of children/adolescents in the household and parental rules for PA were directly and negatively associated with MVPA at school. Whilst these associations were not in the expected direction, there are several possible explanations. For example, the number of children in the household may be an indicator of SES that is not captured by household income. Lower SES households tend to have more children, and this may result in less parental attention and resources being available for each child, compared with households with fewer children [59]. Given their more limited resources [59], parents in lower SES households may also be more likely to send their adolescent children to a local school that may not be particularly supportive of PA. Parents with stricter PA rules may choose schools that have stricter rules about students’ activities and behaviours including the ability to engage in free play or other forms of PA during recess. For example, some larger schools restrict access to their sports/PA equipment, making it available only to their elite sports teams [57].

Neighbourhood SES was positively associated with MVPA at school, possibly due to schools in more affluent areas having better PA facilities, than those in poorer areas. Only two household/family characteristics were directly associated with MVPA after school: parental education (positive association) and parental LTPA (negative association). Compared with less educated parents, those who are more educated may place more value on their adolescent children being active after school. However, another Hong Kong study suggests that after school PA is encouraged as long as study time is not compromised [60].

Strengths of our study include the exploration of complex pathways that link variables at household/family, school and individual levels with school-based PA among adolescents, using a large sample with self-reported PA data and a sub-sample with objectively-measured MVPA. A limitation is that our measure of social support for PA from friends/siblings is not specific to the school setting and this support may occur elsewhere (e.g., at home or in the neighbourhood), however it is likely to include peer/sibling support at school. Another limitation was that student-reported data on PA policies and PA equipment at school were not validated with school documented policies and an audit of PA equipment, and this is recommended for future studies. A further limitation is the inability to determine how much MVPA after school hours was school based. Concurrent Global Positioning Systems (GPS) data would be required to ascertain the location of MVPA after school hours. Future studies of adolescents’ PA should, therefore, consider gathering accelerometer and GPS data simultaneously.

Adolescence is a key developmental life-stage regarding physical psychosocial health and wellbeing, with many competing influences on health behaviours. Given that longitudinal cohort studies have demonstrated that unhealthy behaviours such as physical inactivity and related cardiovascular risk factors during adolescence tend to continue and/or worsen in adulthood [61, 62], it is important to understand the pathways between the above variables to help identify points of intervention. Key points of intervention identified by our study may be in the re-design of PE classes so that adolescents spend more time being physically active during these classes, and promotion of active play during recess. However, further research is required in the Hong Kong setting and should examine additional school-based factors that may potentially influence school-based PA, for example, availability of specialised PE teachers and indoor/outdoor space for PA, as well as the programming of classes and activity breaks. There is a need to identify which options for school sports teams and school-based physical activity would be considered enjoyable by adolescents (especially girls) in Hong Kong, as their enjoyment of PA was strongly associated with PA after school. Future research may include school-based observational studies of PA during PE classes and in the school-yard during recess, to guide the design of PA interventions.

## Conclusions

This study has demonstrated the importance of examining the various pathways through which household/family characteristics, school-based policy and environment, and the characteristics of individual adolescents may impact on how physically activite adolescents are in the school setting. Our findings suggest that the family environment (such as, parental social support for PA) may influence substantially the choice of schools that are more PA-friendly, which, in turn positively impacts on adolescents’ school-based PA as well as on adolescents’ psychosocial characteristics (especially enjoyment of PA) supportive of an active lifestyle.

A complex network of potential pathways of influence on adolescents’ school-based PA was identified. Overall, most of the significant effects were indirect ones and, in some cases, pathways were gender-specific highlighting the need to develop targeted interventions for girls who were less active than boys. The lack of significant pathways between household/family characteristics and objectively-measured MVPA at school compared with self-reported PA at school suggest a mis-match in parental expectation regarding PA levels at school. These findings have implications for the redesign of PE classes to increase PA levels during class and for changes to school policy so that active play is promoted during recess and options for school-based sports teams/programs conducted after school hours are considered by adolescents (particularly girls) to be enjoyable.

Further observational research is required in the school setting in Hong Kong to guide the development of school-based PA interventions.

## Supplementary Information

**Additional file 1. **Family, school and individual characteristics associated with adolescents’ physical activity at school in Hong Kong: the iHealt(H) study. **Table S1.** Estimation of effects of household / family characteristics on school characteristics and the moderating effects of proximity to school and adolescent’s gender (Step 1 of mediation analyses). **Table S2.** Estimation of effects of household / family characteristics and school characteristics on adolescent’s characteristics and the moderating effects of adolescent’s gender (Step 2 of mediation analyses). **Table S3.** Effects of household / family characteristics, school characteristics and adolescent’s characteristics on adolescent’s physical activity at school, and the moderating effects of adolescent’s gender (Step 3 of mediation analysis). **Table S4.** Estimation of effects of household / family characteristics on school characteristics and the moderating effects of proximity to school and adolescent’s gender for sub-sample^a^ (Step 1 of mediation analyses). **Table S5.** Estimation of effects of household / family characteristics and school characteristics on adolescent’s characteristics and the moderating effects of adolescent’s gender for sub-sample^a^ (Step 2 of mediation analyses). **Table S6.** Effects of household / family characteristics, school characteristics and adolescent’s characteristics on adolescent’s objectively-measured physical activity, and the moderating effects of adolescent’s gender for sub-sample^a^ (Step 3 of mediation analysis).

## Data Availability

As the consent forms signed by participants indicated that the data would be only accessible to the team of investigators, the data are confidential.
