# Supplementary material for: Family, school and individual characteristics associated with adolescents’ physical activity at school in Hong Kong: the iHealt(H) study
Source: Int J Behav Nutr Phys Act. 2021 Jan 19;18:14. doi: 10.1186/s12966-021-01085-z (PMC7816388; doi:10.1186/s12966-021-01085-z)
Supplement: Supplementary file 1 — Additional file 1. Family, school and individual characteristics associated with adolescents’ physical activity at school in Hong Kong: the iHealt(H) study. Table S1. Estimation of effects of household / family characteristics on school characteristics and the moderating effects of proximity to school and adolescent’s gender (Step 1 of mediation analyses). Table S2. Estimation of effects of household / family characteristics and school characteristics on adolescent’s characteristics and the moderating effects of adolescent’s gender (Step 2 of mediation analyses). Table S3. Effects of household / family characteristics, school characteristics and adolescent’s characteristics on adolescent’s physical activity at school, and the moderating effects of adolescent’s gender (Step 3 of mediation analysis). Table S4. Estimation of effects of household / family characteristics on school characteristics and the moderating effects of proximity to school and adolescent’s gender for sub-samplea (Step 1 of mediation analyses). Table S5. Estimation of effects of household / family characteristics and school characteristics on adolescent’s characteristics and the moderating effects of adolescent’s gender for sub-samplea (Step 2 of mediation analyses). Table S6. Effects of household / family characteristics, school characteristics and adolescent’s characteristics on adolescent’s objectively-measured physical activity, and the moderating effects of adolescent’s gender for sub-samplea (Step 3 of mediation analysis). [file 12966_2021_1085_MOESM1_ESM.docx]

**Additional file 1 : Family, school and individual characteristics associated with adolescents’ physical activity at school in Hong Kong: the iHealt(H) study**

**Detailed description of analytical steps**

Descriptive statistics were computed for all variables. To estimate how family-level and school-level factors, and adolescent’s individual characteristics were related to adolescent’s PA in the school setting in Hong Kong, and to identify potential mediators of these associations, generalized additive mixed models (GAMMs [1]) were used. The analytical approach followed the assumptions of the model presented in Figure 1 (see manuscript), which hypothesised that (1) the associations of household / family characteristics with adolescent’s PA in the school setting would be partially mediated by school and adolescent’s characteristics, (2) the associations of school characteristics with adolescent’s school-based PA would be partially mediated by adolescent’s characteristics, (3) proximity of school and adolescent’s gender would moderate the associations of household / family characteristics with school characteristics, and (4) adolescent’s gender would moderate the associations of school characteristics with adolescent’s characteristics and school-based PA.

The presence of mediation effects was examined using the joint-significance test [2] and following the steps outlined in Tables S1-S3 for the full sample and Table S4-S6 for the sub-sample who wore accelerometers. According to this test, mediation is confirmed if the associations (regression coefficients) between an exposure and its mediator(s), and the exposure-adjusted associations between the mediator(s) and the outcome are statistically significant. Step 1 of the mediation analysis involved examining the total effects of each of the household / family characteristics on each of our two school characteristic outcomes (i.e., a combined measure of access to PA equipment and PA-friendly school policy; and social support for PA) (Steps 1a to 1k in Table S1 (Table S4, sub-sample)). In addition, in Step 1 of the analyses, proximity of home to school and adolescent’s gender were each examined as potential moderators of associations between household / family characteristics and school characteristics. The theoretical reasons underpinning these moderation analyses are given in the ‘Data analytic plan and hypotheses’ section of the manuscript.

Step 2 of the mediation analysis (Table S2, full sample; Table S5, sub-sample) entailed regressing household / family characteristics and school characteristics on adolescent’s characteristics to estimate the direct effects of school social and physical environmental variables and household characteristics on adolescent’s characteristics. Here, the moderating effects of adolescent’s gender on the associations between school and adolescent’s characteristics were also examined. In Step 3 (Table S3, full sample; Table S6, sub-sample) we examined the direct effects of household / family, school and adolescent characteristics on PA outcomes at school and the moderating effects of adolescent’s gender on the associations between school characteristics and school-based PA. All GAMMs were adjusted for potential confounders identified using directed acyclic graphs as detailed in Tables S1-S6. All analyses were conducted in R version 3.6.3 [3] using the packages ‘mgcv’ version 1.8.31 [4] and ‘multcomp’ version 1.4.13 [5].

**References**

1. Wood S. Generalised additive models: an introduction with R. 2nd ed. Boca Raton, FL: Chapman & Hall/CRC; 2006.

2. MacKinnon DP, Luecken LJ. How and for whom? Mediation and moderation in health psychology. Health Psychol. 2008;27(2S):S99-S100.

3. R Core Team. R: A language and environment for statistical computing. Vienna, Austria: R Foundation for Statistical Computing; 2020.

4. Wood S. Stable and efficient multiple smoothing parameter estimation for generalized additive models. Journal of the American Statistical Association. 2004;99:673-86.

5. Hothorn T, Bretz F, Westfall P. Simultaneous Inference in General Parametric Models. Biometrical Journal 2008;50(3):346-63.

**Table S1. Estimation of effects of household / family characteristics on school characteristics and the moderating effects of proximity to school and adolescent’s gender (Step 1 of mediation analyses)**

|  |  |  | **Regression models** | | | |
| --- | --- | --- | --- | --- | --- | --- |
| **Step** | **Effect estimated** | **Covariate(s)** | **School PA-friendly index^#^** | | **Social support for PA from peers/siblings** | |
|  | |  | *b* (95% CI) | p | e*^b^* (95% CI) | p |
| 1a* | Total effects of highest educational attainment in the household on two school characteristics | None | 0.06 (-0.01,0.12) | 0.082 | **1.05 (1.02,1.09)** | **0.005** |
|  | Moderating effects of gender on the association between highest educational attainment in the household and two school characteristics |  | 0.07 (-0.04,0.19) | 0.210 | 1.02 (0.95.1.09) | 0.577 |
|  | Moderating effects of *proximity of home to school* on the association between highest educational attainment in the household and two school characteristics |  | -0.02 (-0.07,0.03) | 0.414 | **0.97 (0.94,1.00)** | **0.047** |
| 1b* | Total effects of motor vehicles in the household on two school characteristics | Age-parent  Education-parent | **0.14 (0.02,0.26)** | **0.027** | 0.97 (0.90,1.04) | 0.369 |
|  | Moderating effects of gender on the association between motor vehicles in the household and two school characteristics | Marital status  No of children  Neighborhood SES | -0.17 (-0.39,0.06) | 0.153 | 0.93 (0.81,1.07) | 0.301 |
|  | Moderating effects of *proximity of home to school* on the association between motor vehicles in the household and two school characteristics | Self-Selection PA | **-0.11(-0.20, -0.02)** | **0.012** | **0.95 (0.89,1.00)** | **0.047** |
| 1c* | Total effects of number of children on two school characteristics | Age-parent  Education-parent | 0.05 (-0.06,0.16) | 0.393 | **1.20 (1.12,1.28)** | **<0.001** |
|  | Moderating effects of gender on the association between number of children and two school characteristics | Marital status  Motor Vehicle | -0.09 (-0.31,0.12) | 0.388 | 0.97 (0.85,1.10) | 0.632 |
|  | Moderating effects of *proximity of home to school* on the association between number of children and two school characteristics | Neighborhood SES  Self-Selection PA | 0.02 (-0.06,1.00) | 0.595 | 1.03 (0.98,1.08) | 0.207 |
| 1d* | Total effects of neighborhood self-selection related to PA on two school characteristics | Age-parent  Education-parent | 0.07 (-0.04,0.18) | 0.193 | 1.06 (0.99,1.13) | 0.116 |
|  | Moderating effects of gender on the association between neighborhood self-selection related to PA and two school characteristics | Marital status  No of children  Motor Vehicle | **-0.21 (-0.42,0.00)** | **0.054** | **0.86 (0.75,0.98)** | **0.024** |
|  | Moderating effects of *proximity of home to school* on the association between neighborhood self-selection related to PA and two school characteristics | Neighborhood SES | -0.01 (-0.10,0.07) | 0.728 | 1.01 (0.95,1.06) | 0.835 |
| 1e* | Total effects of neighborhood socio-economic status on two school characteristics | Income Household | 0.04 (-0.22,0.29) | 0.777 | 1.12 (0.99,1.26) | 0.079 |
|  | Moderating effects of gender on the association between neighborhood socio-economic status and two school characteristics |  | 0.20 (-0.13,0.53) | 0.235 | 0.91 (0.75,1.12) | 0.386 |
|  | Moderating effects of *proximity of home to school* on the association between neighborhood socio-economic status and two school characteristics |  | 0.03 (-0.12, 0.18) | 0.705 | 0.98 (0.90,1.07) | 0.672 |
| 1f* | Total effects of household income on two school characteristics | Age-parent  Education-parent | -0.02 (-0.05,0.02) | 0.437 | 1.00 (0. 97,1.02) | 0.816 |
|  | Moderating effects of gender on the association between household income and two school characteristics | Marital status | 0.05 (-0.01,0.11) | 0.127 | 1.00 (0.97,1.04) | 0.916 |
|  | Moderating effects of *proximity of home to school* on the association between household income and two school characteristics |  | -0.02 (-0.04,0.01) | 0.210 | 1.00 (0.99,1.02) | 0.748 |
| 1g* | Total effects of parental rules for PA on two school characteristics | Education-parent | 0.01 (-0.01,0.03) | 0.311 | **1.04 (1.02,1.05)** | **<0.001** |
|  | Moderating effects of gender on the association between parental rules for PA and two school characteristics |  | 0.01 (-0.03,0.06) | 0.525 | 0.99 (0.97,1.02) | 0.661 |
|  | Moderating effects of *proximity of home to school* on the association between parental rules for PA and two school characteristics |  | 0.01 (-0.01,0.03) | 0.311 | 1.00 (0.99, 1.01) | 0.988 |
| 1h* | Total effects of social support for PA from parents on two school characteristics | Education-parent  Motor Vehicle | **0.15 (0.06,0.25)** | **0.001** | **1.45 (1.37,1.54)** | **<0.001** |
|  | Moderating effects of gender on the association between social support for PA from parents and two school characteristics | PA equipment home  Leisure PA^ | 0.12 (-0.05,0.29) | 0.180 | 0.98 (0.88,1.09) | 0.711 |
|  | Moderating effects of *proximity of home to school* on the association between social support for PA from parents and two school characteristics |  | 0.03 (-0.03,0.10) | 0.332 | 0.99 (0.95,1.03) | 0.611 |
| 1i* | Total effects of PA equipment at home/neighborhood on two school characteristics | Age-parent  Education-parent | **0.05 (0.01,0.08)** | **0.007** | **1.07 (1.05,1.09)** | **<0.001** |
|  | Moderating effects of gender on the association between PA equipment at home/neighborhood and two school characteristics | Marital status  No of children  Motor Vehicle | 0.01 (-0.05,0.08) | 0.660 | 1.02 (0.98,1.06) | 0.362 |
|  | Moderating effects of *proximity of home to school* on the association between PA equipment at home / neighborhood and two school characteristics | Self-Selection PA | -0.02 (-0.05,0.01) | 0.143 | **0.98 (0.97,1.00)** | **0.026** |
| 1j* | Total effects of parental leisure-time PA^ on two school characteristics | Education-parent  Motor Vehicle | 0.01 (-0.00,0.03) | 0.133 | **1.02 (1.01,1.03)** | **<0.001** |
|  | Moderating effects of gender on the association between parental leisure-time PA^ and two school characteristics | PA equipment home  Self-Selection PA | 0.00 (-0.04,0.04) | 0.919 | **1.02 (1.00,1.04)** | **0.055** |
|  | Moderating effects of *proximity of home to school* on the association between parental leisure-time PA^ and two school characteristics |  | -0.01 (-0.02,0.00) | 0.207 | 1.00 (0.99,1.01) | 0.543 |
| 1k* | Total effects of parental transportation PA^ on two school characteristics | Education-parent  Motor Vehicle | 0.00 (-0.01,0.02) | 0.726 | 1.01 (0.99,1.02) | 0.274 |
|  | Moderating effects of gender on the association between parental transportation PA^ and two school characteristics |  | 0.03 (-0.01,0.06) | 0.122 | 1.00 (0.98,1.02) | 0.873 |
|  | Moderating effects of *proximity of home to school* on the association between parental transportation PA^ and two school characteristics |  | -0.01 (-0.03,0.00) | 0.073 | 1.00 (0.99,1.01) | 0.390 |

^ minutes converted to hours (by dividing by 60); *b* = regression coefficient; CI = confidence interval; e*^b^* = exponentiated regression coefficient; PA = physical activity; ^#^ consisting of items measuring PA equipment at school and school PA-friendly policy supporting after-school PA..

* The moderating effects of adolescent’s gender and/or proximity of home to school on exposure-outcome or mediator-outcome associations were examined by adding two-way interaction terms to these models.

**Table S2. Estimation of effects of household / family characteristics and school characteristics on adolescent’s characteristics and the moderating effects of adolescent’s gender (Step 2 of mediation analyses)**

|  |  |  | **Regression models** | | | | | |
| --- | --- | --- | --- | --- | --- | --- | --- | --- |
| **Step** | **Effect estimated** | **Covariate(s)** | **Attitude towards PA** | | **Enjoyment of PA** | | **Athletic ability** | |
|  | |  | *b* (95% CI) | p | e*^b^* (95% CI) | p | *b* (95% CI) | p |
| 2a* | Direct effects of highest educational attainment in the household on adolescent’s characteristics | Motor Vehicle  H’hold Income  Leisure PA^  N’hood SES | -0.01 (-0.03,0.01) | 0.201 | 0.99 (0.98,1.00) | 0.203 | 0.00 (-0.04,0.05) | 0.888 |
|  | Moderating effects of gender on the association between highest educational attainment in the household and adolescent’s characteristics | Self-selection PA  PA equipment at home  Parental rules PA  Soc Sup from parents  School PA-friendly index^#^  Soc Sup from peers  Transport PA^ | 0.00 (-0.03,0.03) | 0.993 | 1.00 (0.98,1.02) | 0.885 | 0.03 (-0.04,0.11) | 0.387 |
| 2b* | Direct effects of number of motor vehicles in the household on adolescent’s characteristics | Education-parent  H’hold Income  Leisure PA^  Self-selection PA | -0.01 (-0.05,0.02) | 0.412 | 0.99 (0.96,1.01) | 0.201 | -0.01 (-0.09,0.08) | 0.901 |
|  | Moderating effects of gender on the association between number of motor vehicles in the household and adolescent’s characteristics | N’hood SES  PA equipment at home  Parental rules PA  Soc Sup from parents  School PA-friendly index^#^  Soc Sup from peers  Transport PA^ | -0.05 (-0.11,0.01) | 0.122 | 0.98 (0.94,1.02) | 0.299 | 0.15 (-0.00,0.30) | 0.056 |
| 2c* | Direct effects of number of children on adolescent’s characteristics | Education-parent  Motor vehicle  H’hold income | 0.01 (-0.02,0.04) | 0.410 | 1.00 (0.98,1.02) | 0.864 | 0.01 (-0.06,0.09) | 0.739 |
|  | Moderating effects of gender on the association between number of children and adolescent’s characteristics | Leisure PA^  Marital status  PA equipment at home  Self-selection PA | -0.05 (-0.11,0.01) | 0.132 | **0.95 (0.91,0.98)** | **0.006** | -0.06 (-0.21,0.09) | 0.441 |
| 2d* | Direct effects of neighborhood self-selection related to PA on adolescent’s characteristics | Motor Vehicle  Education-parent  H’hold Income | **0.04 (0.01,0.07)** | **0.007** | 1.02 (1.00,1.04) | 0.088 | -0.03 (-0.10,0.05) | 0.490 |
|  | Moderating effects of gender on the association between neighborhood self-selection related to PA and adolescent’s characteristics | Leisure PA^ N’hood SES  PA equipment at home  Parental rules PA  Soc Sup from parents  School PA-friendly index^#^  Soc Sup from peers  Transport PA^ | -0.02 (-0.07,0.04) | 0.551 | 1.00 (0.96,1.04) | 0.993 | -0.12 (-0.27,0.02) | 0.100 |
| 2e* | Direct effects of neighborhood socio-economic status on adolescent’s characteristics | Motor Vehicle  Education-parent  H’hold Income | 0.01 (-0.05,0.07) | 0.685 | 1.02 (0.98,1.05) | 0.337 | -0.01 (-0.13,0.11) | 0.875 |
|  | Moderating effects of gender on the association between neighborhood socio-economic status and adolescent’s characteristics | Leisure PA^  Self-selection PA  PA equipment at home  Parental rules PA  Soc Sup from parents  School PA-friendly index^#^  Soc Sup from peers  Transport PA^ | 0.02 (-0.06,0.11) | 0.612 | 1.03 (0.97,1.09) | 0.343 | 0.05 (-0.17,0.27) | 0.677 |
| 2f* | Direct effects of household income on adolescent’s characteristics | Motor Vehicle  Education-parent  Leisure PA^ | 0.01 (-0.00,0.02) | 0.085 | 1.00 (0.99,1.01) | 0.713 | 0.00 (-0.02,0.03) | 0.752 |
|  | Moderating effects of gender on the association between household income and adolescent’s characteristics | N’hood SES  Self-selection PA  PA equipment at home  Parental rules PA  Soc Sup from parents  School PA-friendly index^#^  Soc Sup from peers  Transport PA^ | -0.01 (-0.02,0.01) | 0.368 | 1.00 (0.99,1.01) | 0.634 | -0.01 (-0.05,0.03) | 0.631 |
| 2g* | Direct effects of parental rules for PA on adolescent’s characteristics | Motor Vehicle  Education-parent  H’hold income | 0.00 (-0.01,0.01) | 0.956 | 1.00 (0.99,1.00) | 0.421 | -0.01 (-0.02,0.01) | 0.396 |
|  | Moderating effects of gender on the association between parental rules for PA and adolescent’s characteristics | Leisure PA^  N’hood SES  Self-selection PA  PA equipment at home  Soc Sup from parents  School PA-friendly index^#^  Soc Sup from peers  Transport PA^ | -0.00 (-0.01,0.01) | 0.693 | 0.99 (0.98,1.00) | 0.181 | -0.02 (-0.05,0.01) | 0.252 |
| 2h* | Direct effects of social support for PA from parents on adolescent’s characteristics | Motor Vehicle  Education-parent  H’hold income | **0.06 (0.03,0.09)** | **<0.001** | **1.04 (1.02,1.06)** | **<0.001** | **0.18 (0.11,0.25)** | **<0.001** |
|  | Moderating effects of gender on the association between social support for PA from parents and adolescent’s characteristics | Leisure PA^  N’hood SES  Self-selection PA  PA equipment at home  Parental rules PA  School PA-friendly index^#^  Soc Sup from peers  Transport PA^ | **-0.05 (-0.09, -0.00)** | **0.038** | 0.98 (0.95,1.01) | 0.207 | -0.07 (-0.18,0.05) | 0.276 |
| 2i* | Direct effects of PA equipment at home / neighborhood on adolescent’s characteristics | Motor Vehicle  Education-parent  H’hold income | 0.00 (-0.01,0.01) | 0.896 | **1.01 (1.00,1.02)** | **0.001** | **0.04 (0.01,0.06)** | **0.003** |
|  | Moderating effects of gender on the association between PA equipment at home / neighborhood and adolescent’s characteristics | Leisure PA^  N’hood SES  Self-selection PA  Parental rules PA  Soc Sup from parents  School PA-friendly index^#^  Soc Sup from peers  Transport PA^ | -0.02 (-0.03,0.00) | 0.057 | 1.00 (0.99,1.01) | 0.991 | -0.00 (-0.05,0.04) | 0.919 |
| 2j* | Direct effects of parental leisure-time PA^ on adolescent’s characteristics | Motor Vehicle  Education-parent  H’hold income | 0.00 (-0.00,0.01) | 0.115 | 1.00 (1.00,1.01) | 0.293 | **0.02 (0.01,0.03)** | **0.002** |
|  | Moderating effects of gender on the association between parental leisure-time PA^ and adolescent’s characteristics | N’hood SES  Self-selection PA  PA equipment at home  Parental rules PA  Soc Sup from parents  School PA-friendly index^#^  Soc Sup from peers  Transport PA^ | -0.01 (-0.02,0.00) | 0.295 | 1.00 (0.99,1.00) | 0.467 | -0.01 (-0.04,0.01) | 0.376 |
| 2k* | Direct effects of parental transport PA^ on adolescent’s characteristics | Motor Vehicle  Education-parent  H’hold income | -0.00(-0.01,0.00) | 0.381 | 1.00 (0.99,1.00) | 0.458 | 0.00 (-0.01,0.01) | 0.878 |
|  | Moderating effects of gender on the association between parental transport PA^ and adolescent’s characteristics | Leisure PA^  N’hood SES  Self-selection PA  PA equipment at home  Parental rules PA  Soc Sup from parents  School PA-friendly index^#^  Soc Sup from peers | 0.00 (-0.01,0.01) | 0.931 | 1.00 (0.99,1.00) | 0.281 | -0.01 (-0.03,0.01) | 0.344 |
| **Effects of school characteristics on adolescent’s characteristics** | | |  |  |  |  |  |  |
| 2l* | Total effects of school PA-friendly index^#^ on adolescent’s characteristics | Motor Vehicle  Education-parent  H’hold income | **0.03 (0.01,0.04)** | **<0.001** | **1.02 (1.01,1.03)** | **<0.001** | **0.06 (0.02,0.09)** | **0.003** |
|  |  | Leisure PA^  N’hood SES  Self-selection PA  PA equipment at home  Parental rules PA  Soc Sup from parents  Transport PA^ |  |  |  |  |  |  |
|  | Direct effects of school PA-friendly index^#^ on adolescent’s characteristics | Motor Vehicle  Education-parent  H’hold income | **0.03 (0.01,0.04)** | **<0.001** | **1.02 (1.01,1.03)** | **<0.001** | **0.05 (0.01,0.09)** | **0.006** |
|  | Moderating effects of gender on the association between school PA-friendly index^#^ and adolescent’s characteristics | Leisure PA^  N’hood SES  Self-selection PA  PA equipment at home  Parental rules PA  Soc Sup from parents  Soc Sup from peers  Transport PA^ | **-0.03 (-0.06, -0.00)** | **0.036** | 0.99 (0.97,1.01) | 0.341 | -0.01 (-0.08,0.07) | 0.804 |
| 2m* | Direct effects of social support from peers on adolescent’s characteristics | Motor Vehicle  Education-parent  H’hold income | **0.06 (0.04,0.08)** | **<0.001** | **1.05 (1.03,1.06)** | **<0.001** | **0.14 (0.08,0.20)** | **<0.001** |
|  | Moderating effects of gender on the association between social support from peers and adolescent’s characteristics | Leisure PA^  N’hood SES  Self-selection PA  PA equipment at home  Parental rules PA  Soc Sup from parents  School PA-friendly index^#^  Transport PA^ | **-0.05 (-0.09, -0.01)** | **0.020** | **0.96 (0.94,0.99)** | **0.007** | 0.03 (-0.07,0.13) | 0.573 |

^ minutes converted to hours (by dividing by 60); *b* = regression coefficient; CI = confidence interval; e*^b^* = exponentiated regression coefficient. PA = physical activity; * The moderating effects of adolescent’s gender on exposure-outcome or mediator-outcome associations were examined by adding two-way interaction terms to these models. ^#^ consisting of items measuring PA equipment at school and school PA-friendly policy supporting after-school PA.

**Table S3. Effects of household / family characteristics, school characteristics and adolescent’s characteristics on** **adolescent’s physical activity at school, and the moderating effects of adolescent’s gender (Step 3 of mediation analysis)**

|  |  |  | **Regression models** | | | |
| --- | --- | --- | --- | --- | --- | --- |
| **Step** | **Effect estimated** | **Covariate(s)** | **PA at school during school hours** | | **PA at school after school hours** | |
| **Household / family characteristics** | |  | *b* (95% CI) | p | e*^b^* (95% CI) | p |
| 3a* | Direct effects of highest educational attainment in the household on PA at school outcomes | Motor vehicle  Household income | 1.57 (-0.48,3.62) | 0.134 | **1.08 (1.01,1.15)** | **0.027** |
|  | Moderating effects of gender on the association between highest educational attainment in the household and PA at school outcomes | Leisure PA^  Neighborhood SES  Self-selection PA  PA equipment home  Parental rule for PA  School PA-friendly index^#^  Social support from adult  Social support from peers  Transport PA^  Attitude towards PA  Enjoyment of PA  Athletic ability | **3.44 (0.20,6.67)** | **0.037** | 0.98 (0.88,1.09) | 0.728 |
| 3b* | Direct effects of motor vehicles in the household on PA at school outcomes | Education-parent  Household income | **4.08 (0.63,7.54)** | **0.021** | 1.11 (1.00,1.24) | 0.054 |
|  | Moderating effects of gender on the association between motor vehicles in the household and PA at school outcomes | Leisure PA^  Neighborhood SES  Self-selection PA  PA equipment home  Parental rule for PA  School PA-friendly index^#^  Social support from adult  Social support from peers  Transport PA^  Attitude towards PA  Enjoyment of PA  Athletic ability | **6.29 (-0.19,12.77)** | **0.057** | 1.12 (0.92,1.37) | 0.267 |
| 3c* | Direct effects of number of children on PA at school outcomes | Education-parent  Motor vehicle | 2.24 (-0.91,5.39) | 0.164 | 1.09 (0.97,1.22) | 0.164 |
|  | Moderating effects of gender on the association between number of children and PA at school outcomes | Household income  Leisure PA^  Marital status  Self-selection PA  PA equipment home | 1.11 (-5.02,7.24) | 0.723 | 1.12 (0.89,1.40) | 0.327 |
| 3d* | Direct effects of neighborhood self-selection related to PA on PA at school outcomes | Education-parent  Motor vehicle | -1.76 (-4.83,1.31) | 0.262 | 0.94 (0.84,1.04) | 0.202 |
|  | Moderating effects of gender on the association between neighborhood self-selection related to PA and PA at school outcomes | Household income  Leisure PA^  Neighborhood SES  PA equipment home  Parental rule for PA  School PA-friendly index^#^  Social support from adult  Social support from peers  Transport PA^  Attitude towards PA  Enjoyment of PA  Athletic ability | 2.55 (-3.42,8.53) | 0.402 | 1.03 (0.85,1.26) | 0.734 |
| 3e* | Direct effects of neighborhood socio-economic status on PA at school outcomes | Education-parent  Motor vehicle | **17.77(9.27,26.27)** | **<0.001** | 1.11 (0.91,1.37) | 0.305 |
|  | Moderating effects of gender on the association between neighborhood socio-economic status and PA at school outcomes | Household income  Leisure PA^  Self-selection PA  PA equipment home  Parental rule for PA  School PA-friendly index^#^  Social support from adult  Social support from peers  Transport PA^  Attitude towards PA  Enjoyment of PA  Athletic ability | 8.68 (-0.57,17.93) | 0.066 | **1.38 (1.02,1.86)** | **0.035** |
| 3f* | Direct effects of household income on PA at school outcomes | Education-parent  Motor vehicle | **1.15 (0.06,2.24)** | **0.039** | 0.99 (0.96,1.03) | 0.601 |
|  | Moderating effects of gender on the association between household income and PA at school outcomes | Leisure PA^  Neighborhood SES  Self-selection PA  PA equipment home  Parental rule for PA  School PA-friendly index^#^  Social support from adult  Social support from peers  Transport PA^  Attitude towards PA  Enjoyment of PA  Athletic ability | 1.58 (-0.12,3.29) | 0.069 | 1.02 (0.96,1.08) | 0.513 |
| 3g* | Direct effects of parental rules for PA on PA at school outcomes | Education-parent  Motor vehicle | 0.41 (-0.23,1.05) | 0.206 | **1.02 (1.00,1.04)** | **0.040** |
|  | Moderating effects of gender on the association between parental rules for PA and PA at school outcomes | Household income  Leisure PA^  Neighborhood SES  Self-selection PA  PA equipment home  School PA-friendly index^#^  Social support from adult  Social support from peers  Transport PA^  Attitude towards PA  Enjoyment of PA  Athletic ability | 0.70 (-0.52,1.92) | 0.261 | 1.00 (0.96,1.04) | 0.941 |
| 3h* | Direct effects of social support for PA from parents on PA at school outcomes | Education-parent  Motor vehicle | 1.04 (-1.84,3.91) | 0.479 | 1.05 (0.96,1.15) | 0.308 |
|  | Moderating effects of gender on the association between social support for PA from parents and PA at school outcomes | Household income  Leisure PA^  Neighborhood SES  Self-selection PA  PA equipment home  Parental rule for PA  School PA-friendly index^#^  Social support from peers  Transport PA^  Attitude towards PA  Enjoyment of PA  Athletic ability | **5.03 (0.20,9.85)** | **0.041** | 1.04 (0.89,1.22) | 0.623 |
| 3i* | Direct effects of PA equipment at home / neighborhood on PA at school outcomes | Education-parent  Motor vehicle | 0.26 (-0.72,1.23) | 0.607 | **1.05 (1.02,1.09)** | **0.003** |
|  | Moderating effects of gender on the association between PA equipment at home / neighborhood and PA at school outcomes | Household income  Leisure PA^  Neighborhood SES  Self-selection PA  Parental rule for PA  School PA-friendly index^#^  Social support from adult  Social support from peers  Transport PA^  Attitude towards PA  Enjoyment of PA  Athletic ability | 0.42 (-1.42,2.26) | 0.654 | 1.02 (0.96,1.08) | 0.566 |
| 3j* | Direct effects of parental leisure time PA^ on PA at school outcomes | Education-parent  Motor vehicle | **0.62 (0.12,1.12)** | **0.016** | 1.01 (1.00,1.03) | 0.083 |
|  | Moderating effects of gender on the association between parental leisure time PA^ and PA at school outcomes | Household income  Neighborhood SES  Self-selection PA  PA equipment home  Parental rule for PA  School PA-friendly index^#^  Social support from adult  Social support from peers  Transport PA^  Attitude towards PA  Enjoyment of PA  Athletic ability | **1.64 (0.55,2.73)** | **0.003** | 0.99 (0.95,1.02) | 0.442 |
| 3k* | Direct effects of parental transportation for PA^ on PA at school outcomes | Education-parent  Motor vehicle | 0.22 (-0.24,0.69) | 0.351 | 1.01 (0.99,1.02) | 0.377 |
|  | Moderating effects of gender on the association between parental transportation for PA^ and PA at school outcomes | Household income  Leisure PA^  Neighborhood SES  Self-selection PA  PA equipment home  Parental rule for PA  School PA-friendly index^#^ Social support from adult  Social support from peers  Attitude towards PA  Enjoyment of PA  Athletic ability | **1.07 (0.14,2.00)** | **0.024** | 1.02 (0.99,1.06) | 0.133 |
| **School characteristics** | |  |  |  |  |  |
| 3l* | Direct effects of school PA-friendly index^#^ on PA at school outcomes | Education-parent  Motor vehicle | **2.76 (1.19,4.33)** | **<0.001** | **1.14 (1.08,1.20)** | **<0.001** |
|  | Moderating effects of gender on the association between school PA-friendly index^#^ and PA at school outcomes | Household income  Leisure PA^  Neighborhood SES  Self-selection PA  PA equipment home  Parental rule for PA  Social support from adult  Social support from peers  Transport PA^  Attitude towards PA  Enjoyment of PA  Athletic ability | -0.26 (-3.34,2.83) | 0.870 | 1.04 (0.94,1.15) | 0.444 |
| 3m* | Direct effects of social support from peers on PA at school outcomes | Education-parent  Motor vehicle | **2.82 (0.43,5.22)** | **0.021** | **1.13 (1.04,1.22)** | **0.003** |
|  | Moderating effects of gender on the association between social support from peers and PA at school outcomes | Household income  Leisure PA^  Neighborhood SES  Self-selection PA  PA equipment home  Parental rule for PA  School PA-friendly index^#^ Social support from adult  Transport PA^  Attitude towards PA  Enjoyment of PA  Athletic ability | -1.47 (-5.72,2.78) | 0.498 | 0.94 (0.82,1.08) | 0.405 |
| **Adolescent’s characteristics** | |  |  |  |  |  |
| 3n* | Direct effects of attitude towards PA on PA at school outcomes | Education-parent  Motor vehicle | **11.13(5.36,16.91)** | **<0.001** | **2.05 (1.66,2.52)** | **<0.001** |
|  | Moderating effects of gender on the association between attitude towards PA and PA at school outcomes | Household income  Leisure PA^  Neighborhood SES  Self-selection PA  PA equipment home  Parental rule for PA  School PA-friendly index^#^  Social support from adult  Social support from peers  Transport PA^ | -0.72(-11.86,10.43) | 0.900 | 0.69 (0.46,1.02) | 0.062 |
| 3o* | Direct effects of enjoyment of PA on PA at school outcomes | Education-parent  Motor vehicle | **4.48(2.21,6.75)** | **<0.001** | **1.56 (1.43,1.69)** | **<0.001** |
|  | Moderating effects of gender on the association between enjoyment of PA and PA at school outcomes | Household income  Leisure PA^  Neighborhood SES  Self-selection PA  PA equipment home  Parental rule for PA  School PA-friendly index^#^ Social support from adult  Social support from peers  Transport PA^ | 2.62 (1.77,7.02) | 0.241 | 0.88 (0.75,1.02) | 0.095 |
| 3p* | Direct effects of athletic ability on PA at school outcomes | Education-parent  Motor vehicle | **5.04 (2.84,7.24)** | **<0.001** | **1.78 (1.65,1.92)** | **<0.001** |
|  | Moderating effects of gender on the association between athletic ability and PA at school outcomes | Household income  Leisure PA^  Neighborhood SES  Self-selection PA  PA equipment home  Parental rule for PA  School PA-friendly index^#^ Social support from adult  Social support from peers  Transport PA^ | 2.19 (-2.05,6.42) | 0.312 | 0.96 (0.83,1.11) | 0.585 |

^ minutes converted to hours (by dividing by 60); *b* = regression coefficient; CI = confidence interval; e*^b^* = exponentiated regression coefficient. PA = physical activity; * The moderating effects of adolescent’s gender on exposure-outcome or mediator-outcome associations were examined by adding two-way interaction terms to these models. ^#^ consisting of items measuring PA equipment at school and school PA-friendly policy supporting after-school PA.

**Table S4. Estimation of effects of household / family characteristics on school characteristics and the moderating effects of proximity to school and adolescent’s gender for sub-sample^a^ (Step 1 of mediation analyses)**

|  |  |  | **Regression models** | | | |
| --- | --- | --- | --- | --- | --- | --- |
| **Step** | **Effect estimated** | **Covariate(s)** | **School PA-friendly index**^#^ | | **Social support for PA from peers/siblings** | |
|  | |  | *b* (95% CI) | p | e*^b^* (95% CI) | p |
| 1a* | Total effects of highest educational attainment in the household on two school characteristics | None | **0.09 (-0.00,0.19)** | **0.051** | **1.09 (1.03,1.15)** | **0.002** |
|  | Moderating effects of gender on the association between highest educational attainment in the household and two school characteristics |  | **0.18 (0.01,0.35)** | **0.036** | 1.07 (0.96,1.18) | 0.204 |
|  | Moderating effects of *proximity of home to school* on the association between highest educational attainment in the household and two school characteristics |  | 0.04 (-0.03,0.11) | 0.300 | 0.97 (0.93,1.01) | 0.170 |
| 1b* | Total effects of motor vehicles in the household on two school characteristics | Age-parent  Education-parent | 0.11 (-0.06,0.28) | 0.212 | 0.91 (0.82,1.01) | 0.090 |
|  | Moderating effects of gender on the association between motor vehicles in the household and two school characteristics | Marital status  No of children  Neighborhood SES | -0.19 (-0.53,0.15) | 0.267 | 0.99 (0.80,1.22) | 0.915 |
|  | Moderating effects of *proximity of home to school* on the association between motor vehicles in the household and two school characteristics | Self-Selection PA | -0.10 (-0.23,0.03) | 0.119 | **0.90 (0.82,0.98)** | **0.015** |
| 1c* | Total effects of number of children on two school characteristics | Age-parent  Education-parent | 0.01 (-0.16,0.18) | 0.909 | **1.23 (1.11,1.36)** | **<0.001** |
|  | Moderating effects of gender on the association between number of children and two school characteristics | Marital status  Motor Vehicle | 0.07 (-0.26,0.39) | 0.682 | 0.97 (0.80,1.17) | 0.73 |
|  | Moderating effects of *proximity of home to school* on the association between number of children and two school characteristics | Neighborhood SES  Self-Selection PA | 0.00 (-0.12,0.12) | 0.987 | 1.00 (0.93,1.06) | 0.887 |
| 1d* | Total effects of neighborhood self-selection related to PA on two school characteristics | Age-parent  Education-parent | -0.04 (-0.21,0.12) | 0.607 | 1.01 (0.91,1.12) | 0.877 |
|  | Moderating effects of gender on the association between neighborhood self-selection related to PA and two school characteristics | Marital status  No of children  Motor Vehicle | -0.12 (0.45,0.21) | 0.468 | **0.81 (0.66,0.99)** | **0.045** |
|  | Moderating effects of *proximity of home to school* on the association between neighborhood self-selection related to PA and two school characteristics | Neighborhood SES | -0.03 (-0.16,0.10) | 0.692 | 1.00 (0.92,1.08) | 0.921 |
| 1e* | Total effects of neighborhood socio-economic status on two school characteristics | Income Household | 0.19 (-0.17,0.55) | 0.306 | 1.16 (0.96,1.41) | 0.128 |
|  | Moderating effects of gender on the association between neighborhood socio-economic status and two school characteristics |  | 0.44 (-0.05,0.93) | 0.077 | 0.75 (0.56,1.01) | 0.062 |
|  | Moderating effects of *proximity of home to school* on the association between neighborhood socio-economic status and two school characteristics |  | -0.02 (-0.23,0.19) | 0.831 | 0.96 (0.85,1.08) | 0.460 |
| 1f* | Total effects of household income on two school characteristics | Age-parent  Education-parent | -0.03 (-0.09,0.03) | 0.282 | 0.98 (0.95,1.02) | 0.371 |
|  | Moderating effects of gender on the association between household income and two school characteristics | Marital status | 0.06 (-0.03,0.15) | 0.204 | 0.99 (0.93,1.04) | 0.659 |
|  | Moderating effects of *proximity of home to school* on the association between household income and two school characteristics |  | 0.01 (-0.03,0.05) | 0.649 | 1.00 (0.97,1.02) | 0.753 |
| 1g* | Total effects of parental rules for PA on two school characteristics | Education-parent | -0.02 (-0.05,0.01) | 0.262 | **1.03 (1.01,1.05)** | **0.007** |
|  | Moderating effects of gender on the association between parental rules for PA and two school characteristics |  | 0.01 (-0.06,0.08) | 0.775 | 0.97 (0.93,1.01) | 0.134 |
|  | Moderating effects of *proximity of home to school* on the association between parental rules for PA and two school characteristics |  | 0.00 (-0.02,0.03) | 0.774 | 1.00 (0.98,1.01) | 0.827 |
| 1h* | Total effects of social support for PA from parents on two school characteristics | Education-parent  Motor Vehicle | **0.17 (0.04,0.31)** | **0.013** | **1.41 (1.30,1.53)** | **<0.001** |
|  | Moderating effects of gender on the association between social support for PA from parents and two school characteristics | PA equipment home  Leisure PA^ | 0.23 (-0.02,0.48) | 0.071 | 1.00 (0.86,1.16) | 0.987 |
|  | Moderating effects of *proximity of home to school* on the association between social support for PA from parents and two school characteristics |  | -0.00 (-0.10,0.09) | 0.938 | 0.98 (0.92,1.04) | 0.428 |
| 1i* | Total effects of PA equipment at home/neighborhood on two school characteristics | Age-parent  Education-parent | 0.05 (-0.01,0.10) | 0.079 | **1.06 (1.02,1.09)** | **<0.001** |
|  | Moderating effects of gender on the association between PA equipment at home/neighborhood and two school characteristics | Marital status  No of children  Motor Vehicle | 0.06 (-0.04,0.16) | 0.270 | **1.08 (1.02,1.15)** | **0.014** |
|  | Moderating effects of *proximity of home to school* on the association between PA equipment at home / neighborhood and two school characteristics | Self-Selection PA | -0.01 (-0.05,0.03) | 0.679 | 0.99 (0.97,1.01) | 0.367 |
| 1j* | Total effects of parental leisure-time PA^ on two school characteristics | Education-parent  Motor Vehicle | 0.00 (-0.03,0.03) | 0.998 | **1.02 (1.01,1.04)** | **0.007** |
|  | Moderating effects of gender on the association between parental leisure-time PA^ and two school characteristics | PA equipment home  Self-Selection PA | -0.01 (-0.07,0.05) | 0.844 | **1.03 (1.00,1.07)** | **0.058** |
|  | Moderating effects of *proximity of home to school* on the association between parental leisure-time PA^ and two school characteristics |  | 0.00 (-0.02,0.02) | 0.856 | 1.00 (0.99,1.01) | 0.667 |
| 1k* | Total effects of parental transportation PA^ on two school characteristics | Education-parent  Motor Vehicle | -0.01 (-0.03,0.02) | 0.531 | 1.00 (0.99,1.02) | 0.704 |
|  | Moderating effects of gender on the association between parental transportation PA^ and two school characteristics |  | 0.02 (-0.03,0.07) | 0.523 | 0.98 (0.95,1.01) | 0.303 |
|  | Moderating effects of *proximity of home to school* on the association between parental transportation PA^ and two school characteristics |  | -0.01 (-0.03,0.01) | 0.320 | **1.01 (1.00,1.02)** | **0.045** |

^a^ sub-sample of adolescents who wore accelerometer; ^ minutes converted to hours (by dividing by 60); *b* = regression coefficient.

CI = confidence interval; e*^b^* = exponentiated regression coefficient; PA = physical activity; ^#^ consisting of items measuring PA equipment at school and school PA-friendly policy supporting after-school PA; * The moderating effects of adolescent’s gender and/or proximity of home to school on exposure-outcome or mediator-outcome associations were examined by adding two-way interaction terms to these models.

**Table S5. Estimation of effects of household / family characteristics and school characteristics on adolescent’s characteristics and the moderating effects of adolescent’s gender for sub-sample^a^ (Step 2 of mediation analyses)**

|  |  |  | **Regression models** | | | | | |
| --- | --- | --- | --- | --- | --- | --- | --- | --- |
| **Step** | **Effect estimated** | **Covariate(s)** | **Attitude towards PA** | | **Enjoyment of PA** | | **Athletic performance** | |
|  | |  | *b* (95% CI) | p | e*^b^* (95% CI) | p | *b* (95% CI) | p |
| 2a* | Direct effects of highest educational attainment in the household on adolescent’s characteristics | Motor Vehicle  H’hold Income  Leisure PA^  N’hood SES | -0.00 (-0.03,0.03) | 0.891 | 1.00 (0.98,1.02) | 0.840 | 0.06 (-0.02,0.13) | 0.133 |
|  | Moderating effects of gender on the association between highest educational attainment in the household and adolescent’s characteristics | Self-selection PA  PA equipment at home  Parental rules PA  Soc Sup from parents  School PA-friendly index^#^  Soc Sup from peers  Transport PA^ | -0.00 (-0.05,0.04) | 0.917 | 1.00 (0.97,1.03) | 0.959 | **0.11 (-0.01,0.23)** | **0.063** |
| 2b* | Direct effects of number of motor vehicles in the household on adolescent’s characteristics | Education-parent  H’hold Income  Leisure PA^  Self-selection PA | -0.02 (-0.07,0.03) | 0.382 | 0.99 (0.96,1.03) | 0.678 | -0.04 (-0.16,0.08) | 0.536 |
|  | Moderating effects of gender on the association between number of motor vehicles in the household and adolescent’s characteristics | N’hood SES  PA equipment at home  Parental rules PA  Soc Sup from parents  School PA-friendly index^#^  Soc Sup from peers  Transport PA^ | -0.03 (-0.12,0.06) | 0.529 | 1.00 (0.97,1.06) | 0.995 | 1.00 (-0.14,0.33) | 0.414 |
| 2c* | Direct effects of number of children on adolescent’s characteristics | Education-parent  Motor vehicle  H’hold income | 0.01 (-0.04,0.05) | 0.761 | 1.00 (0.97,1.03) | 0.972 | 0.02 (-0.09,0.14) | 0.703 |
|  | Moderating effects of gender on the association between number of children and adolescent’s characteristics | Leisure PA^  Marital status  PA equipment at home  Self-selection PA | **-0.09 (-0.18, -0.00)** | **0.044** | **0.90 (0.85,0.96)** | **<0.001** | -0.12 (-0.35,0.10) | 0.278 |
| 2d* | Direct effects of neighborhood self-selection related to PA on adolescent’s characteristics | Motor Vehicle  Education-parent  H’hold Income | **0.05 (0.01,0.10)** | **0.028** | 1.00 (0.97,1.03) | 0.998 | -0.05(-0.17,0.06) | 0.381 |
|  | Moderating effects of gender on the association between neighborhood self-selection related to PA and adolescent’s characteristics | Leisure PA^ N’hood SES  PA equipment at home  Parental rules PA  Soc Sup from parents  School PA-friendly index^#^  Soc Sup from peers  Transport PA^ | -0.02 (-0.11,0.07) | 0.638 | 1.00 (0.95,1.07) | 0.881 | 0.01 (-0.22,0.23) | 0.964 |
| 2e* | Direct effects of neighborhood socio-economic status on adolescent’s characteristics | Motor Vehicle  Education-parent  H’hold Income | -0.01 (-0.11,0.09) | 0.813 | 0.99 (0.94,1.05) | 0.779 | -0.01(-0.21,0.19) | 0.897 |
|  | Moderating effects of gender on the association between neighborhood socio-economic status and adolescent’s characteristics | Leisure PA^  Self-selection PA  PA equipment at home  Parental rules PA  Soc Sup from parents  School PA-friendly index^#^  Soc Sup from peers  Transport PA^ | 0.05 (-0.09,0.18) | 0.498 | 1.03 (0.94,1.12) | 0.564 | 0.03 (-0.31,0.36) | 0.876 |
| 2f* | Direct effects of household income on adolescent’s characteristics | Motor Vehicle  Education-parent  Leisure PA^ | **0.02 (0.00,0.03)** | **0.030** | 1.00 (0.99,1.01) | 0.488 | 0.01 (-0.03,0.05) | 0.674 |
|  | Moderating effects of gender on the association between household income and adolescent’s characteristics | N’hood SES  Self-selection PA  PA equipment at home  Parental rules PA  Soc Sup from parents  School PA-friendly index^#^  Soc Sup from peers  Transport PA^ | -0.00 (-0.03,0.02) | 0.747 | 1.00 (0.98,1.01) | 0.809 | -0.01 (-0.07,0.05) | 0.713 |
| 2g* | Direct effects of parental rules for PA on adolescent’s characteristics | Motor Vehicle  Education-parent  H’hold income | 0.00 (-0.01,0.01) | 0.873 | 1.00 (0.99,1.01) | 0.652 | -0.01(-0.03,0.02) | 0.463 |
|  | Moderating effects of gender on the association between parental rules for PA and adolescent’s characteristics | Leisure PA^  N’hood SES  Self-selection PA  PA equipment at home  Soc Sup from parents  School PA-friendly index^#^  Soc Sup from peers  Transport PA^ | **0.02 (0.00,0.04)** | **0.032** | 1.00 (0.99,1.01) | 0.926 | -0.02 (-0.07,0.02) | 0.348 |
| 2h* | Direct effects of social support for PA from parents on adolescent’s characteristics | Motor Vehicle  Education-parent  H’hold income | **0.05 (0.01,0.09)** | **0.021** | 1.02 (0.99,1.05) | 0.174 | **0.15 (0.04,0.25)** | **0.006** |
|  | Moderating effects of gender on the association between social support for PA from parents and adolescent’s characteristics | Leisure PA^  N’hood SES  Self-selection PA  PA equipment at home  Parental rules PA  School PA-friendly index^#^  Soc Sup from peers  Transport PA^ | -0.03 (-0.10,0.04) | 0.399 | 1.00 (0.95,1.05) | 0.959 | -0.06 (-0.24,0.11) | 0.475 |
| 2i* | Direct effects of PA equipment at home / neighborhood on adolescent’s characteristics | Motor Vehicle  Education-parent  H’hold income | -0.00 (-0.02,0.01) | 0.718 | 1.01 (1.00,1.02) | 0.119 | **0.04 (0.00,0.08)** | **0.032** |
|  | Moderating effects of gender on the association between PA equipment at home / neighborhood and adolescent’s characteristics | Leisure PA^  N’hood SES  Self-selection PA  Parental rules PA  Soc Sup from parents  School PA-friendly index^#^  Soc Sup from peers  Transport PA^ | -0.01 (-0.04,0.02) | 0.384 | 1.01 (0.99,1.03) | 0.282 | -0.02 (-0.09,0.05) | 0.617 |
| 2j* | Direct effects of parental leisure-time PA^ on adolescent’s characteristics | Motor Vehicle  Education-parent  H’hold income | 0.00 (-0.00,0.01) | 0.279 | 1.00 (1.00,1.01) | 0.101 | 0.01 (-0.01,0.03) | 0.222 |
|  | Moderating effects of gender on the association between parental leisure-time PA^ and adolescent’s characteristics | N’hood SES  Self-selection PA  PA equipment at home  Parental rules PA  Soc Sup from parents  School PA-friendly index^#^  Soc Sup from peers  Transport PA^ | -0.00 (-0.02,0.02) | 0.935 | 1.00 (0.99,1.01) | 0.786 | -0.01 (-0.05,0.03) | 0.618 |
| 2k* | Direct effects of parental transport PA^ on adolescent’s characteristics | Motor Vehicle  Education-parent  H’hold income | -0.01 (-0.01,0.00) | 0.141 | 1.00 (0.99,1.00) | 0.569 | 0.01 (-0.01,0.02) | 0.507 |
|  | Moderating effects of gender on the association between parental transport PA^ and adolescent’s characteristics | Leisure PA^  N’hood SES  Self-selection PA  PA equipment at home  Parental rules PA  Soc Sup from parents  School PA-friendly index^#^  Soc Sup from peers | -0.01 (-0.02,0.00) | 0.127 | **0.98 (0.97,0.99)** | **<0.001** | -0.03 (-0.06,0.01) | 0.128 |
| **Effects of school characteristics on adolescent’s characteristics** | | |  |  |  |  |  |  |
| 2l* | Total effects of school PA-friendly index^#^ on adolescent’s characteristics | Motor Vehicle  Education-parent  H’hold income | 0.01 (-0.01,0.03) | 0.337 | **1.02 (1.01,1.04)** | **0.003** | 0.01 (-0.05,0.07) | 0.707 |
|  |  | Leisure PA^  N’hood SES  Self-selection PA  PA equipment at home  Parental rules PA  Soc Sup from parents  Transport PA^ |  |  |  |  |  |  |
|  | Direct effects of school PA-friendly index^#^ on adolescent’s characteristics | Motor Vehicle  Education-parent  H’hold income | 0.01 (-0.01,0.03) | 0.472 | **1.02 (1.01,1.04)** | **0.006** | 0.01 (-0.05,0.06) | 0.842 |
|  | Moderating effects of gender on the association between school PA-friendly index^#^ and adolescent’s characteristics | Leisure PA^  N’hood SES  Self-selection PA  PA equipment at home  Parental rules PA  Soc Sup from parents  Soc Sup from peers  Transport PA^ | -0.04 (-0.08,0.01) | 0.128 | 0.99 (0.96,1.02) | 0.439 | -0.09 (-0.20,0.03) | 0.136 |
| 2m* | Direct effects of social support from peers on adolescent’s characteristics | Motor Vehicle  Education-parent  H’hold income | **0.06 (0.03,0.10)** | **<0.001** | **1.04 (1.02,1.06)** | **<0.001** | **0.15 (0.06,0.23)** | **<0.001** |
|  | Moderating effects of gender on the association between social support from peers and adolescent’s characteristics | Leisure PA^  N’hood SES  Self-selection PA  PA equipment at home  Parental rules PA  Soc Sup from parents  School PA-friendly index^#^  Transport PA^ | -0.01 (-0.07,0.05) | 0.720 | **0.96 (0.93,1.00)** | **0.070** | 0.10 (-0.06,0.25) | 0.209 |

^a^ sub-sample of adolescents who wore accelerometer; ^ minutes converted to hours (by dividing by 60); *b* = regression coefficient.

CI = confidence interval; e*^b^* = exponentiated regression coefficient; PA = physical activity.

* The moderating effects of adolescent’s gender on exposure-outcome or mediator-outcome associations were examined by adding two-way interaction terms to these models. ^#^ consisting of items measuring PA equipment at school and school PA-friendly policy supporting after-school PA.

**Table S6. Effects of household / family characteristics, school characteristics and adolescent’s characteristics on adolescent’s objectively-measured physical activity, and the moderating effects of adolescent’s gender for sub-sample^a^ (Step 3 of mediation analysis)**

|  |  |  | **Regression models** | | | |
| --- | --- | --- | --- | --- | --- | --- |
| **Step** | **Effect estimated** | **Covariate(s)** | **Average MVPA (min/day) during school hours** | | **Average MVPA (min/day) after school hours** | |
| **Household / family characteristics** | |  | e*^b^* (95% CI) | p | e*^b^* (95% CI) | p |
| 3a* | Direct effects of highest educational attainment in the household on PA at school outcomes | Motor vehicle  Household income | 1.02 (0.98,1.06) | 0.399 | **1.06 (1.01,1.11)** | **0.029** |
|  | Moderating effects of gender on the association between highest educational attainment in the household and PA at school outcomes | Leisure PA^  Neighborhood SES  Self-selection PA  PA equipment home  Parental rule for PA  School PA-friendly index^#^  Social support from adult  Social support from peers  Transport PA^  Attitude towards PA  Enjoyment of PA  Athletic ability | 0.95 (0.90,1.01) | 0.081 | 0.98 (0.91,1.06) | 0.619 |
| 3b* | Direct effects of motor vehicles in the household on PA at school outcomes | Education-parent  Household income | 0.99 (0.93,1.06) | 0.816 | 0.99 (0.92,1.07) | 0.849 |
|  | Moderating effects of gender on the association between motor vehicles in the household and PA at school outcomes | Leisure PA^  Neighborhood SES  Self-selection PA  PA equipment home  Parental rule for PA  School PA-friendly index^#^  Social support from adult  Social support from peers  Transport PA^  Attitude towards PA  Enjoyment of PA  Athletic ability | 0.97 (0.86,1.09) | 0.583 | 1.04 (0.90,1.02) | 0.617 |
| 3c* | Direct effects of number of children on PA at school outcomes | Education-parent  Motor vehicle | **0.93 (0.87,0.99)** | **0.018** | 0.99 (0.93,1.07) | 0.891 |
|  | Moderating effects of gender on the association between number of children and PA at school outcomes | Household income  Leisure PA^  Marital status  Self-selection PA  PA equipment home | 1.06 (0.95,1.18) | 0.336 | 0.91 (0.79,1.05) | 0.188 |
| 3d* | Direct effects of neighborhood self-selection related to PA on PA at school outcomes | Education-parent  Motor vehicle | 0.98 (0.92,1.04) | 0.462 | 1.02 (0.94,1.10) | 0.642 |
|  | Moderating effects of gender on the association between neighborhood self-selection related to PA and PA at school outcomes | Household income  Leisure PA^  Neighborhood SES  PA equipment home  Parental rule for PA  School PA-friendly index^#^  Social support from adult  Social support from peers  Transport PA^  Attitude towards PA  Enjoyment of PA  Athletic ability | 0.99 (0.88,1.10) | 0.822 | 0.90 (0.78,1.04) | 0.144 |
| 3e* | Direct effects of neighborhood socio-economic status on PA at school outcomes | Education-parent  Motor vehicle | **1.35 (1.18,1.54)** | **<0.001** | 1.11 (0.97,1.26) | 0.124 |
|  | Moderating effects of gender on the association between neighborhood socio-economic status and PA at school outcomes | Household income  Leisure PA^  Self-selection PA  PA equipment home  Parental rule for PA  School PA-friendly index^#^  Social support from adult  Social support from peers  Transport PA^  Attitude towards PA  Enjoyment of PA  Athletic ability | 0.89 (0.75,1.05) | 0.164 | 0.95 (0.77,1.18) | 0.643 |
| 3f* | Direct effects of household income on PA at school outcomes | Education-parent  Motor vehicle | 0.99 (0.97,1.01) | 0.337 | 0.99 (0.96,1.01) | 0.309 |
|  | Moderating effects of gender on the association between household income and PA at school outcomes | Leisure PA^  Neighborhood SES  Self-selection PA  PA equipment home  Parental rule for PA  School PA-friendly index^#^  Social support from adult  Social support from peers  Transport PA^  Attitude towards PA  Enjoyment of PA  Athletic ability | 1.01 (0.98,1.05) | 0.360 | 0.98 (0.95,1.02) | 0.444 |
| 3g* | Direct effects of parental rules for PA on PA at school outcomes | Education-parent  Motor vehicle | **0.98 (0.97,0.99)** | **0.005** | 0.99 (0.97,1.00) | 0.084 |
|  | Moderating effects of gender on the association between parental rules for PA and PA at school outcomes | Household income  Leisure PA^  Neighborhood SES  Self-selection PA  PA equipment home  School PA-friendly index^#^  Social support from adult  Social support from peers  Transport PA^  Attitude towards PA  Enjoyment of PA  Athletic ability | 1.01 (0.98,1.03) | 0.560 | 1.00 (0.97,1.03) | 0.865 |
| 3h* | Direct effects of social support for PA from parents on PA at school outcomes | Education-parent  Motor vehicle | 0.99 (0.93,1.04) | 0.605 | 1.03 (0.96,1.10) | 0.399 |
|  | Moderating effects of gender on the association between social support for PA from parents and PA at school outcomes | Household income  Leisure PA^  Neighborhood SES  Self-selection PA  PA equipment home  Parental rule for PA  School PA-friendly index^#^  Social support from peers  Transport PA^  Attitude towards PA  Enjoyment of PA  Athletic ability | 1.02 (0.94,1.11) | 0.624 | 0.92 (0.83,1.03) | 0.140 |
| 3i* | Direct effects of PA equipment at home / neighborhood on PA at school outcomes | Education-parent  Motor vehicle | 0.99 (0.97,1.01) | 0.542 | 0.99 (0.97,1.01) | 0.396 |
|  | Moderating effects of gender on the association between PA equipment at home / neighborhood and PA at school outcomes | Household income  Leisure PA^  Neighborhood SES  Self-selection PA  Parental rule for PA  School PA-friendly index^#^  Social support from adult  Social support from peers  Transport PA^  Attitude towards PA  Enjoyment of PA  Athletic ability | 1.00 (0.96,1.03) | 0.931 | 0.96 (0.92,1.01) | 0.115 |
| 3j* | Direct effects of parental leisure time PA^ on PA at school outcomes | Education-parent  Motor vehicle | 1.00 (0.99,1.01) | 0.655 | **0.99 (0.98,1.00)** | **0.025** |
|  | Moderating effects of gender on the association between parental leisure time PA^ and PA at school outcomes | Household income  Neighborhood SES  Self-selection PA  PA equipment home  Parental rule for PA  School PA-friendly index^#^  Social support from adult  Social support from peers  Transport PA^  Attitude towards PA  Enjoyment of PA  Athletic ability | 0.99 (0.97,1.01) | 0.333 | 0.99 (0.97,1.02) | 0.679 |
| 3k* | Direct effects of parental transportation for PA^ on PA at school outcomes | Education-parent  Motor vehicle | 1.00 (0.99,1.01) | 0.735 | 1.01 (0.99,1.02) | 0.275 |
|  | Moderating effects of gender on the association between parental transportation for PA^ and PA at school outcomes | Household income  Leisure PA^  Neighborhood SES  Self-selection PA  PA equipment home  Parental rule for PA  School PA-friendly index^#^ Social support from adult  Social support from peers  Attitude towards PA  Enjoyment of PA  Athletic ability | 1.01 (0.99,1.02) | 0.507 | 1.02 (0.99,1.04) | 0.154 |
| **School characteristics** | |  |  |  |  |  |
| 3l* | Direct effects of school PA-friendly index^#^ on PA at school outcomes | Education-parent  Motor vehicle | 0.99 (0.96,1.02) | 0.462 | 1.02 (0.98,1.06) | 0.285 |
|  | Moderating effects of gender on the association between school PA-friendly index^#^ and PA at school outcomes | Household income  Leisure PA^  Neighborhood SES  Self-selection PA  PA equipment home  Parental rule for PA  Social support from adult  Social support from peers  Transport PA^  Attitude towards PA  Enjoyment of PA  Athletic ability | 0.96 (0.91,1.02) | 0.163 | **0.93 (0.86,0.99)** | **0.034** |
| 3m* | Direct effects of social support from peers on PA at school outcomes | Education-parent  Motor vehicle | 1.00 (0.95,1.04) | 0.884 | 0.97 (0.92,1.02) | 0.287 |
|  | Moderating effects of gender on the association between social support from peers and PA at school outcomes | Household income  Leisure PA^  Neighborhood SES  Self-selection PA  PA equipment home  Parental rule for PA  School PA-friendly index^#^ Social support from adult  Transport PA^  Attitude towards PA  Enjoyment of PA  Athletic ability | 1.00 (0.92,1.08) | 0.962 | 0.97 (0.88,1.07) | 0.498 |
| **Adolescent’s characteristics** | |  |  |  |  |  |
| 3n* | Direct effects of attitude towards PA on PA at school outcomes | Education-parent  Motor vehicle | 1.13 (0.99,1.29) | 0.073 | 1.09 (0.93,1.28) | 0.275 |
|  | Moderating effects of gender on the association between attitude towards PA and PA at school outcomes | Household income  Leisure PA^  Neighborhood SES  Self-selection PA  PA equipment home  Parental rule for PA  School PA-friendly index^#^  Social support from adult  Social support from peers  Transport PA^ | 1.03 (0.85,1.27) | 0.739 | 1.00 (0.77,1.28) | 0.98 |
| 3o* | Direct effects of enjoyment of PA on PA at school outcomes | Education-parent  Motor vehicle | 1.03 (0.97,1.08) | 0.332 | **1.07 (1.01,1.15)** | **0.032** |
|  | Moderating effects of gender on the association between enjoyment of PA and PA at school outcomes | Household income  Leisure PA^  Neighborhood SES  Self-selection PA  PA equipment home  Parental rule for PA  School PA-friendly index^#^ Social support from adult  Social support from peers  Transport PA^ | 0.99 (0.91,1.07) | 0.781 | 0.96 (0.86,1.06) | 0.436 |
| 3p* | Direct effects of athletic ability on PA at school outcomes | Education-parent  Motor vehicle | 1.04 (0.99,1.09) | 0.104 | 1.03 (0.97,1.09) | 0.373 |
|  | Moderating effects of gender on the association between athletic ability and PA at school outcomes | Household income  Leisure PA^  Neighborhood SES  Self-selection PA  PA equipment home  Parental rule for PA  School PA-friendly index^#^ Social support from adult  Social support from peers  Transport PA^ | 1.05 (0.96,1.13) | 0.274 | 1.02 (0.92,1.13) | 0.664 |

^a^ sub-sample of adolescents who wore accelerometer; ^ minutes converted to hours (by dividing by 60); *b* = regression coefficient.

CI = confidence interval; e*^b^* = exponentiated regression coefficient; PA = physical activity.

* The moderating effects of adolescent’s gender on exposure-outcome or mediator-outcome associations were examined by adding two-way interaction terms to these models. ^#^ consisting of items measuring PA equipment at school and school PA-friendly policy supporting after-school PA.
